# Preparing a Phytosome for Promoting Delivery Efficiency and Biological Activities of Methyl Jasmonate-Treated *Dendropanax morbifera* Adventitious Root Extract (DMARE)

**DOI:** 10.3390/biom14101273

**Published:** 2024-10-10

**Authors:** Fengjiao Xu, Shican Xu, Li Yang, Aili Qu, Dongbin Li, Minfen Yu, Yongping Wu, Shaojian Zheng, Xiao Ruan, Qiang Wang

**Affiliations:** 1 School of Biological and Chemical Engineering, NingboTech University, Ningbo 315100, China; xfj2024@nbt.edu.cn (F.X.); yangli@nbt.edu.cn (L.Y.); quaili@nit.zju.edu.cn (A.Q.); ypwu@nit.net.cn (Y.W.); 2 State Key Laboratory of Plant Environmental Resilience, College of Life Sciences, Zhejiang University, Hangzhou 310058, China; sjzheng@zju.edu.cn; 3 National Key Laboratory of Cotton Bio-Breeding and Integrated Utilization, College of Agriculture, Henan University, Kaifeng 475004, China; xu921812@gmail.com; 4 Ningbo Forest Farm, Ningbo Bureau of Natural Resources and Planning, Ningbo 315440, China; comeonldb@163.com (D.L.); 15957495963@163.com (M.Y.)

**Keywords:** *D. morbifera*, adventitious root, phytosome, anti-inflammation, anti-lung cancer

## Abstract

(1) Background: Methyl jasmonate-treated *D. morbifera* adventitious root extract (MeJA-DMARE), enriched with phenolics, has enhanced bioactivities. However, phenolics possess low stability and bioavailability. Substantial evidence indicates that plant extract–phospholipid complex assemblies, known as phytosomes, represent an innovative drug delivery system. (2) Methods: The phytosome complex was created by combining MeJA-DMARE with Soy-L-α-phosphatidylcholine (PC) using three different ratios through two distinct methods (co-solvency method: A1, A2, and A3; thin-layer film method: B1, B2, and B3). (3) Results: Initial evaluation based on UV-Vis, entrapment efficiency (EE%), and loading content (LC%) indicated that B2 exhibited the highest EE% (79.98 ± 1.45) and LC% (69.17 ± 0.14). The phytosome displayed a spherical morphology with a particle size of 210 nm, a notably low polydispersity index of 0.16, and a superior zeta potential value at −25.19 mV. The synthesized phytosome exhibited superior anti-inflammatory activities by inhibiting NO and ROS production (reduced to 8.9% and 55.1% at 250 μg/mL) in RAW cells and adjusting the expression of related inflammatory cytokines; they also slowed lung tumor cell migration (only 2.3% of A549 cells migrated after treatment with phytosomes at 250 μg/mL), promoting ROS generation in A549 cell lines (123.7% compared to control) and stimulating apoptosis of lung cancer-related genes. (4) Conclusions: In conclusion, the MeJA-DMARE phytosome offers stable, economically efficient, and environmentally friendly nanoparticles with superior inflammation and lung tumor inhibition properties. Thus, the MeJA-DMARE phytosome holds promise as an applicable and favorable creation for drug delivery and lung cancer treatment.

## 1. Introduction

The *Dendropanax* genus, part of the Araliaceae family, is distributed in the southwestern parts of East Asia. In addition, it is a neotropical genus containing about 91 to 95 species found in Peninsular Malaysia and Central and South America [1]. In some places, different parts of certain species can act as useful medicines for treating various diseases [2,3]. It was initially discovered on Jesu, Wando, Geomungo, Haenam, Geoje islands, and the southern shore, and its range has contracted due to forest clearing and habitat degradation from urban disregard; currently, it is only found on some parts of the island [1,4]. Various components of *D. morbifera*, including the consumable leaf, bark, seeds, stems, and roots, are acknowledged as alternatives, traditional remedies, and food supplements, and are listed by the Korean Ministry of Food and Drug Safety [5,6,7,8].

Many reports previously have highlighted that *D. morbifera* possesses anti-oxidant [9], anti-microbial [4], anti-inflammatory [3], anti-amnesic [10], anti-thrombotic [11], anti-atherogenic [12], anticancer [9], and anti-complement activity [13] and induces the removal of mercury residues and toxic cadmium from human serum [14]. The genus *Dendropanax* contains polyphenols, flavonoids, terpenoids, and alkaloids [13]; however, these useful compounds are chemically and physiologically fragile, insoluble in aqueous systems, or have negative side effects. In previous work, MeJA-DMARE with an increased amount of 3,5-DCQA exhibited excellent anti-inflammatory and anti-lung cancer activities compared to DMARE [15]. To address these problems, a phytosome was produced. Phytosomes can be generated by combining 2–3 moles of a natural or synthetic phospholipid with 1 mole of plant extracts. Molar ratios for phospholipids/phytoconstituents can range from 0.5 to 3. The preferred molar ratio, however, is typically given as 1:1 [16].

There are various methods for the production of phytosomes, such as the co-solvency technique and thin-layer film method [17]. In the co-solvency method, the medication and phospholipids are each dissolved in an appropriate solvent. The two are then mixed by gentle stirring until a clear mixture is created. The finished homogenous solution is then vacuum-freeze-dried and kept in an airtight container for later use [18]. It is particularly effective for compounds with poor solubility in water, allowing the active ingredient to dissolve effectively before the formation of the phytosome. However, it may result in lower control over particle size. The thin-layer hydration technique involves dissolving phytoconstituents (PCs) in methanol or dichloromethane. After that, the mixture is evaporated in a rotary evaporator to create a thin, dry layer. After that, distilled water is used to hydrate the film [19]. However, the process is more time-consuming and may require additional steps (such as sonication) to ensure proper dispersion of the phytosome components.

A great deal of research work is underway, and recent studies have shown that phytosome methodology is an innovative approach to enhancing the absorption and bioavailability of plant extracts while notably reducing the required dosage levels. The applicability of the technology and the increased demand for herbal medicine for the management of various diseases in the current climate pave the way for new research [20].

## 2. Materials and Methods

### 2.1. Materials and Chemicals

Soy-L-α-phosphatidylcholine was purchased from Sigma-Merck (Avanti), Alabaster, AL, USA, dichloromethane was provided by Daejung, Siheung-si, Republic of Korea, and cellular tubular membranes MWCO 6000–8000 were bought from CellSep, Dongil Biotech, Seoul-si, Republic of Korea. Glycine, hydrochloric acid, citric acid, sodium phosphate monobasic, sodium phosphate dibasic, Tris-HCl buffer (pH 8.0), sodium chloride, bovine serum albumin, DPPH (2,2-diphenyl-1-picryl-hydrazyl), potassium ferricyanide, trichloroacetic acid, and ferric chloride were purchased from Sigma-Aldrich (Saint Louis, MO, USA). The murine macrophage cell line (RAW 264.7) and the lung cancer cell line (A549) were obtained from the Korea Cell Line Bank (KCLB) in Seoul, Republic of Korea. Dulbecco’s Modified Eagle Medium (DMEM) with L-glutamine, RPMI 1640 culture medium containing L-glutamine, and phosphate-buffered saline (PBS, pH 7.4) were purchased from Welgene Inc., located in Gyeongsan-si, Republic of Korea. Penicillin/streptomycin solution and fetal bovine serum were sourced from GenDEPOT. The 3-(4,5-dimethylthiazol-2-yl)-2,5-diphenyltetrazolium bromide (MTT) was procured from Gibco, Waltham, MA, USA.

### 2.2. Synthesis of Phytosome

#### 2.2.1. Preparation of MeJA-DMARE

**Figure 1:**
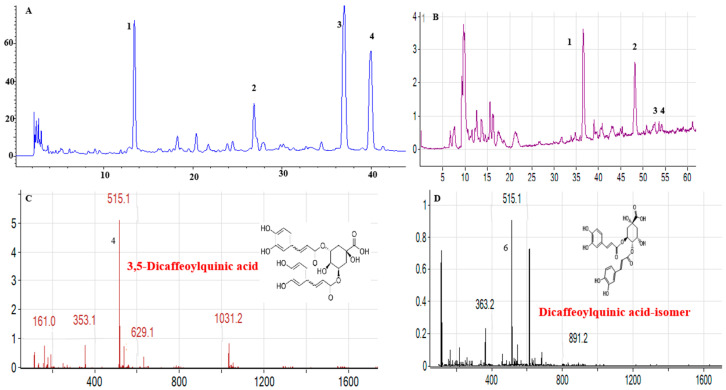
Compounds presented in MeJA-DMARE were analyzed using HPLC and LC-MS. (**A**) Phenolics identified by HPLC; (**B**) phenolic compounds detected by LC-MS; (**C**) LC-MS analysis shows that compound **2** in DMARE corresponds to 3,5-Dicaffeoylquinic acid; (**D**) LC-MS analysis indicates that compound **3** in MeJA-DMARE is a Dicaffeoylquinic acid isomer.

The MeJA-DMARE powder was collected as in our previous studies [15]. The DMARE was desiccated at 60 °C in an oven and then pulverized into a fine powder. Extraction was carried out with 70% ethanol using an ultrasound-assisted method at 80 °C for an hour, as per earlier studies [21]. Subsequently, the extracts were spun at 7000 rpm for a 10 min duration. Following centrifugation, the clear liquid was separated using Whatman filter paper No. 41. This process was conducted three times. The extracts from the 70% ethanol extraction were then concentrated under a rotary evaporator. Once the solvent was completely removed, the resulting powder was gathered and stored at 4 °C for subsequent applications. The composites of MeJA-DMARE were detected in our previous research [15], as shown in Figure 1.

#### 2.2.2. Phytosome Synthesis by Co-Solvency Methods

**Figure 2:**
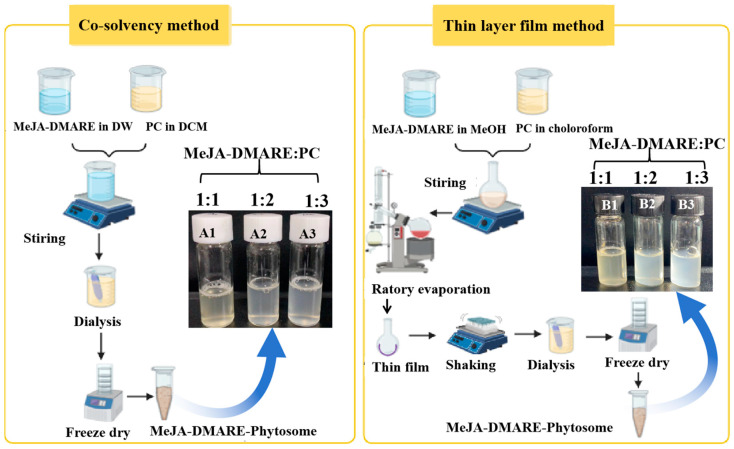
Schematic illustrations of the co-solvency and thin-layer film processes for manufacturing MeJA-DMARE phytosomes. A1, A2, and A3 are the phytosomes synthesized by the co-solvency method; B1, B2, and B3 are the phytosomes prepared by the thin-layer film method.

Phytosome synthesis by the co-solvency method was based on previous research with some modifications [22]. Briefly, 5 mL of distilled water was added to two distinct flasks, and 500 μL of dichloromethane was used to dissolve the PC according to the formulation elucidated in Figure 2. The dissolved PC was incrementally added to the MeJA-DMARE solution and mixed with a magnetic stirrer (Lab companion, Fenton, MO, USA) for 24 h until dichloromethane was completely evaporated and the colloidal solution was formed. After mixing, the solution was collected and dialysis was continued for 1–2 h. It was freeze-dried (Hanil Sci., Gimpo, Republic of Korea) to obtain powder after dialyzing the solution.

#### 2.2.3. Phytosome Synthesis by Thin-Layer Film Method

Phytosome synthesis by the thin-layer film method was based on previous research with some modifications [22]. Briefly, the dried MeJA-DMARE was dissolved in 5 mL methanol and PC in 5 mL chloroform according to the formulation clarified in Figure 2. The mixture was refluxed under a vacuum using a rotary evaporator (Tokyo RIKAKIKAI Co., Ltd., Tokyo, Japan. Made by Eyela) at 30 °C and 120 rpm for 2 h. After the solvents had completely evaporated, the remaining residue was rehydrated with distilled water to create a phytosome suspension. The solution was then sonicated (BRANSON Ultrasonication, EMERSON, Dambury, CT, USA) for 20 min after being put on a shaking machine (N-BIOTE Co., Ltd., Incheon, Republic of Korea) at room temperature and 70 rpm for an hour. After that, the solution was collected and continued with dialysis for 1–2 h. It was freeze-dried (Hanil Sci., Gimpo, Republic of Korea) to obtain powder after dialyzing the solution.

### 2.3. Characterization of Phytosome

#### 2.3.1. UV-Vis

Nanoparticles exhibit interactions with certain light wavelengths, providing them with distinctive optical characteristics. Consequently, the synthesis of MeJA-DMARE phytosomes was monitored by examining the absorbance spectrum of the reaction blend. A UV-Vis spectrophotometer (Ultrospec 2100 pro, GE Healthcare Bio-Sciences Corp., Piscataway, NJ, USA) was applied to read the reaction mixtures at 200–700 nm.

#### 2.3.2. Entrapment Efficiency (EE) and Loading Capacity (LC)

The ultracentrifugation method was used to evaluate the EE and LC of MeJA-DMARE phytosomes [23]. Briefly, 1 mg of MeJA-DMARE phytosome formulations was subjected to centrifugation at 4 °C and 15,000 rpm for 90 min in a refrigerated centrifuge. The amount of MeJA-DMARE was evaluated by measuring the absorbance at λmax = 310 nm with a spectrophotometer (Ultrospec 2100 pro, GE Healthcare Bio-Sciences Corp., Piscataway, NJ, USA) and using a calibration curve (concentration range 0.05–0.6 mg/mL, R2 = 0.998 (*n* = 3)). The absorption was modeled by the following Y = 3.2607X + 0.0305. The following formulations were used to determine EE and LC:

$$EE\%=\frac{Actual\;amount\;of\;MeJA\_DMARE\;in\;phytosome\;formulation}{Theoretical\;amount\;of\;MeJA\_DMARE\;in\;phytosome\;formulation}\times 100\%$$

$$LC\%=\frac{Actual\;amount\;of\;MeJA\_DMARE\;in\;phytosome\;formulation}{Amount\;of\;total\;phytosome}\times 100\%$$

#### 2.3.3. Tyndall Effect

To explore the Tyndall impact on the MeJA-DMARE phytosomes, laser light was used to validate colloidal formation in nanoparticles.

#### 2.3.4. Morphology Study

The phytosome’s form, structure, and arrangement were identified using field emission transmission electron microscopy (FE-TEM) and energy-dispersive X-ray (EDX) mapping with a JEM-2100F instrument from JEOL, Tokyo, Japan, set to 200 kV. For examination, the nanoparticle solution was deposited onto carbon-supported copper grids, then dried at 60 °C to remove any residual solvent. The surface morphology was investigated with a field emission scanning electron microscope (FE-SEM), specifically the LEO SUPRA 55, GENESIS 200 from Carl Zeiss, Jena, Germany, featuring a thermal field emission gun, with resolutions ranging from 1.0 nm at 15 kV to 4.0 nm at 0.1 kV and a magnification spectrum of 12× to 900,000×.

#### 2.3.5. Particle Size, Polydispersity Index, and Zeta Potential

The size distribution and zeta potential of the phytosome in an aqueous environment were evaluated at 25 °C using the ELS-Z2 series particle size analyzer (Otsuka Electronics Co., Osaka, Japan).

#### 2.3.6. Fourier Transform Infrared Spectroscopy (FTIR) Analysis

The MeJA-DMARE phytosomes were subjected to Fourier transform infrared (FTIR) spectroscopy analysis to investigate the functional group interactions on the phytosome surface that act as catalysts and sealing agents. For the FTIR analysis, the dehydrated phytosome powder was analyzed across a wavenumber range of 4000–450 cm^−1^ with a resolution of 4 cm^−1^, utilizing a PerkinElmer Spectrum One FTIR spectrometer [24].

#### 2.3.7. In Vitro Stability Studies

The in vitro stability of the synthesized phytosomes was examined in the presence of the following solutions: distilled water, 20 mM glycine–HCl buffer (pH 2.0), citrate–sodium citrate buffer (pH 5.0), sodium phosphate buffer (pH 7.0), Tris-HCl buffer (pH 8.0), 5% sodium chloride, 10% sodium chloride, and 5% bovine serum albumin. A total of 900 μL of the solvents was combined with 100 μL of the phytosome solution, and the mixture was then incubated at 37 °C. We determined whether there had been a significant change in the surface plasmon wavelength supporting the stability of phytosomes using a UV-Vis spectrophotometer [25].

### 2.4. Evaluation of Biological Activities

#### 2.4.1. Cell Cultures

The A549 cell line was cultivated in 89% RPMI 1640 media, while the murine macrophage cell line (RAW 264.7) was cultured in DMEM. Both cell lines were grown in a moistened atmosphere at 37 °C with 5% CO_2_ in a medium containing 10% FBS and 1% penicillin/streptomycin. They were given 24 h to adhere and develop before exposure to various substances. They were given 24 h to adhere and develop before exposure to various substances.

#### 2.4.2. Cytotoxicity Assay

For cytotoxicity analysis, cells were individually plated into a 96-well plate at a density of 2 × 10^4^ cells per well and then cultured in a 37 °C environment with 5% CO_2_ for a period of 24 h. Cytotoxicity was assayed after 24-h exposure to MeJA-DMARE, the MeJA-DMARE phytosome complex, and PC at various concentrations (31.25, 62.5, 125, 250, 500 μg/mL). The cells were thoroughly washed three times with PBS after discarding the supernatant. Then, the cells were treated with 20 μL of MTT (5 mg/mL in PBS) and incubated for 3 h at 37 °C. The supernatant was discarded and 100 μL of DMSO was added to each well to dissolve the insoluble formazan under dark conditions. A blank was set by adding DMSO to the plate. The baseline reading was removed from the experimental readings. The percentage of cellular viability was determined by the following calculation: Viability % = (Sample Absorbance/Control Absorbance) × 100. Absorbance at 570 nm for each well was detected with a plate reader (Bio Tek Instruments, Inc., Winooski, VT, USA).

#### 2.4.3. Inhibition of Nitric Oxide (NO) Production

RAW 264.7 cells were initially treated with MeJA-DMARE and MeJA-DMARE phytosomes at different dilutions for 1 h. LPS (1 µg/mL) was used to activate the cells, followed by a 24 h incubation period. The nitrite level was determined using Grise’s reagent. Briefly, absorbance was measured at 540 nm with a microplate reader (BioTek Instruments, Inc., Winooski, VT, USA) after mixing Gries’s reagent and supernatant (100 μL, 100 μL). In this experiment, a positive control (standard inhibitor) at 50 μM was set using L-NG-Monomethylarginine Acetate Salt (L-NMMA). The data were presented as NO production (%) in triplicates.

#### 2.4.4. Reactive Oxygen Species (ROS) Generation Assay

The amount of ROS in A549 cells was assessed using the chemical 2′,7′-dichloro-dihydro-fluorescein diacetate (DCFH-DA). A549 cells were plated at a density of 1 × 10^4^ cells per well in a 96-well plate and subsequently maintained at 37 °C with 5% CO_2_ for a 24 h period. After seeding, the cells were treated for 24 h to 125 µg/mL of MeJA-DMARE and MeJA-DMARE phytosomes for ROS generation. Conversely, RAW 264.7 cells were exposed to the test substances in conjunction with LPS to induce an inflammatory response. Following a 24 h period of incubation in a serum-deprived environment, the cells underwent a triple washing with PBS. Subsequently, they were incubated with 20 µM DCFH-DA for half an hour at 37 °C, shielded from light. The spent medium was removed, and the cells were subjected to a double wash with 100 µL of PBS. The generation of reactive oxygen species (ROS) was quantified using a multi-mode plate reader (fluorescence spectrometer), assessing fluorescence intensity at excitation and emission wavelengths of 485 nm and 528 nm, respectively.

#### 2.4.5. The Analysis of Inflammation and Cancer-Related Gene Expression Levels

The total RNA was extracted from the RAW 264.7 and A549 cell lines using the QIAzol lysis reagent (QIAGEN, Germantown, MD, USA). The amfifiRivert reverse transcription kit (GenDepot, Barker, TX, USA) was used following the manufacturer’s instructions to create cDNA by adding 1 µg of extracted RNA to a reaction mixture of 20 µL. The following temperatures were used to create cDNA: 25 °C for 5 min, 42 °C for 59 min, and 70 °C for 15 min. qRT-PCR was carried out using Enzynomics’ SYBR TOPreal qPCR 2X Premix, available in Daejeon, Republic of Korea. In summary, the reactions required a final volume of 10 µL, consisting of 2x Master Mix, 1 µL of template cDNA, and 1 µL each of forward and reverse primers. They were run in triplicate. All real-time measurements were conducted with the CFX Connect Real-Time PCR system (Bio-Rad, Hercules, CA, USA). For the amplification processes, the following conditions were used: 95 °C for 10 min, followed by 40 cycles of 95 °C for 20 s and 55 °C for 30 s, followed by 72 °C for 15 s. The GAPDH gene was used to normalize the data after measuring the relative amounts of mRNAs using the comparative 2^−ΔΔCt^ technique.

#### 2.4.6. Wound-Healing Assay

The motility of A549 cells was tested by conducting a scratch assay. In a 6-well plate, the A549 cells were seeded at 2 × 10^4^ cells per well and kept for 24 h at 37 °C. A vertical scratch was then made in the cell monolayer using a 10 µL sterile pipette tip, and PBS was used to wash away detached cells. The cells were then treated with 250 µg/mL of MeJA-DMARE and MeJA-DMARE phytosomes. Three days into the treatment, documentation was performed using a 5.0-megapixel MC 170 HD camera, sourced from Wetzlar, Germany.

### 2.5. Statistical Analysis

Analysis of the collected data was performed with the aid of GraphPad 9.0 software (GraphPad Software, San Diego, CA, USA). For experimental data obtained in vitro, comparisons of the average results were made via one-way ANOVA, followed by Dunnett’s multiple comparisons test. Each test was replicated at least thrice for consistency, unless noted differently, and the levels of significance were marked as follows: * for *p* < 0.05, ** for *p* < 0.01, *** for *p* < 0.001, and # for *p* < 0.05, ## for *p* < 0.01, ### for *p* < 0.001.

## 3. Results and Discussions

### 3.1. Formulation of MeJA-DMARE Phytosome Complex

The MeJA-DMARE phytosomes were prepared by the co-solvency and thin-layer film methods (Figure 2).

### 3.2. Characterization of Phytosome

#### 3.2.1. Consistency Analysis of Bioactive Components

**Figure 3:**
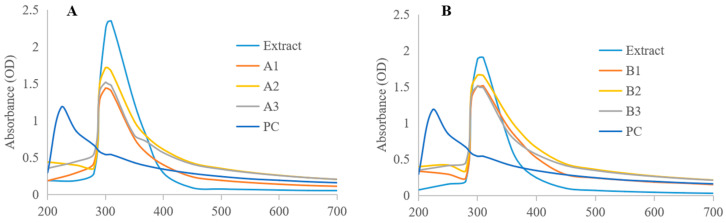
UV-Vis absorbance spectra of MeJA-DMARE phytosomes. The surface plasmons in the MeJA-DMARE phytosomes are shown at ~310 nm. (**A**) MeJA-DMARE phytosome synthesized by the co-solvency method; (**B**) MeJA-DMARE phytosome synthesized by the thin-layer film method.

The visible absorption bands of MeJA-DMARE phytosomes were observed by UV-Vis spectroscopy. The optical characteristics of the phytosomes are extremely sensitive to variations in size, shape, agglomeration, and concentration. Surface plasmon polariton resonances (SPPRs), induced by electromagnetic radiation, are responsible for the collective oscillations of conduction electrons, which impart nanoparticles with their distinctive optical properties. The refractive index close to the phytosome’s surface is affected by those changes, making it possible to describe nanomaterials using UV-Vis spectroscopy [26]. Phosphatidylcholine (PC) exhibits a characteristic absorption peak at 224 nm, while MeJA-DMARE displays a maximum absorption peak at 310 nm, as shown in Figure 3. The complex also shows a maximum absorption peak at 310 nm, although its peak value is lower than that of MeJA-DMARE. This indicates that the chemical structure of the complex remains unchanged and primarily exhibits the chemical properties of MeJA-DMARE. The slight decrease in the peak value suggests that the characteristic structure might be participating in the binding of MeJA-DMARE to phospholipids.

#### 3.2.2. Entrapment Efficiency (EE) and Loading Capacity (LC)

The EE% and LC% of six phytosome formulations were determined, as depicted in Table 1. EE% is a crucial physicochemical characteristic in the creation of nanovesicles. It displays the proportion of drugs entrapped in a complex (phytosome) against the total drug added. The EE% values were variable because the mole ratio of MeJA-DMARE to phosphatidylcholine varied among the six phytosomes. MeJA-DMARE phytosomes with higher ratios gave higher EE% in both synthesis methods, which showed similar results to the previous study [27]. The outcome can be attributed to the notion that an increase in PC within the formulation leads to a higher number of PC molecules available to engage with the phytochemicals present in MeJA-DMARE. Furthermore, PC provides numerous binding sites for phytoconstituents, facilitating the capture of additional phytoconstituents. [28]. From six formulas that have been prepared in Table 1, the B2 formula prepared by the thin-layer film method gave the highest EE% (79.98 ± 1.45) and LC% (69.17 ± 0.14), which did not show a significant difference with A2 produced by the co-solvency method (EE% and LC% were 71.19 ± 4.88 and 62.91 ± 3.11, respectively). One possible explanation is that 1 mol of natural or synthetic phospholipids can achieve the best encapsulation efficiency by capturing 2 mol of plant extract. The thin-layer film method offered more possibilities for the adequate reaction of phytochemicals and PC by rotating at a certain temperature.

#### 3.2.3. Molecular Characterization of Phytosomes

**Figure 4:**
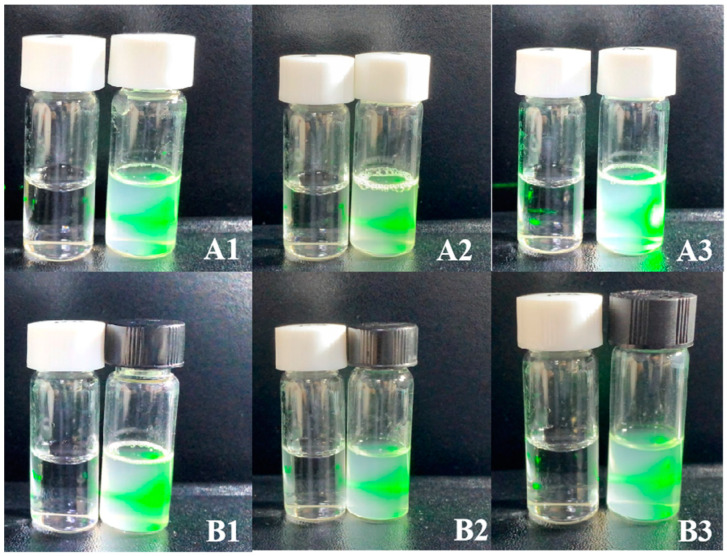
Tyndall effects of MeJA-DMARE phytosomes. (**A1**–**A3**) are the Tyndall effects of phytosomes by the co-solvency method in 3 formulations; (**B1**–**B3**) are the Tyndall effects of phytosomes by the thin-layer film method in 3 formulations.

The Tyndall effect is an intriguing phenomenon that frequently occurs in colloidal solutions. The SPPR signaling may allow for quick qualitative (or semi-quantitative) analysis with the naked eye as well as precise quantitative assessment with a UV-Vis spectrometer. Recent research has shown that the Tyndall effect offers much-improved signaling sensitivity compared to the widely used SPPR method and strong universality for designing a variety of sensing platforms with either colored or colorless colloidal probes just using a smartphone and a laser pointer pen [29,30]. The visualization and Tyndall effect of the synthesized phytosomes are depicted in Figure 4. These images demonstrate that the phytosome particles were evenly distributed, which was confirmed visually as colloids, especially B2. Thus, B2, with the highest loading content and entrapment efficiency, was used for further characterization.

**Figure 5:**
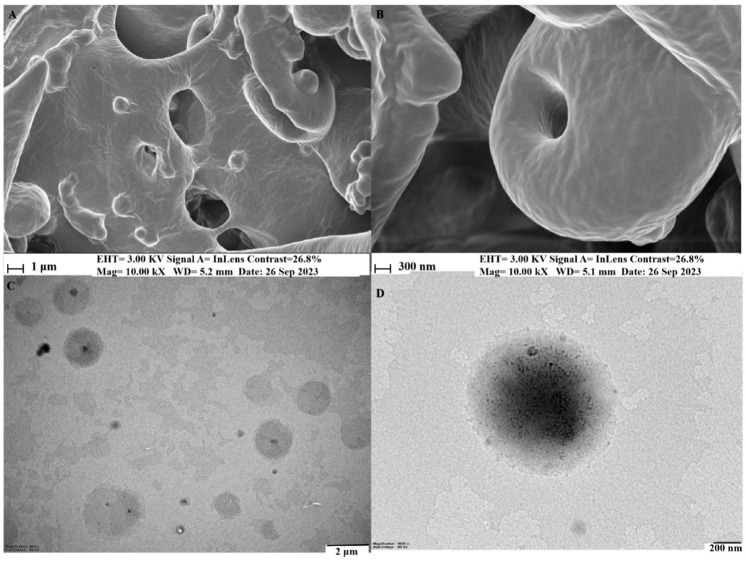
Structural and morphological characterization by (**A**,**B**) FE-SEM and (**C**,**D**) FE-TEM images of MeJA-DMARE phytosomes.

SEM and TEM analyses revealed liposome-like vesicles with an incorporated structure, even size, and well-distributed dispersion in a water atmosphere. The transmission electron micrograph depicted a spherical–translucent morphology with a self-closed, rough surface, and no signs of particle aggregation. Figure 5 displays the surface morphology, shape, and structure of MeJA-DMARE phytosomes. MeJA-DMARE forms spherical structures when associated with phospholipids.

Dynamic light scattering (DLS) relies on the principle of Brownian motion exhibited by dispersed particles. These particles exhibit random motion in a liquid medium, constantly interacting with the surrounding solvent molecules through collisions. These collisions facilitate the transfer of energy, subsequently prompting particle movement. The transferred energy remains relatively consistent and typically exerts a more distinct impact on smaller particles. Consequently, smaller particles tend to move at higher speeds than larger particles. As a result, DLS enables the determination of a particle’s hydrodynamic diameter within a solution [31].

Particle size and its distribution represent crucial parameters for nanoparticles, given their profound influence on various aspects, including drug release kinetics, bio-distribution, mucoadhesive properties, cellular uptake in aqueous environments, buffer exchange within the nanoparticles, and protein diffusion [32,33]. To achieve optimal clinical outcomes, it is essential to have microspheres characterized by a narrow and uniform size distribution [34,35]. Furthermore, achieving a uniform size distribution is crucial for maintaining strong physical stability. The surface charge of a suspension or dispersion determines zeta potential. Understanding this characteristic can be useful in forecasting and managing the behavior of phytosome nanoparticles in vivo [36].

Particle size distribution is typically determined by measuring the polydispersity index (PDI), which can also be assessed using dynamic light scattering (DLS) [37]. PDI values between 0 and 0.5 indicate a monodisperse and homogeneous particle system; however, values exceeding 0.5 suggest non-homogeneity and polydispersity. A PDI value of less than 0.3 is generally considered a homogeneous solid lipid-based nanoparticle (SLBN) [38,39].

Zeta potential represents the total charge of particles in a particular medium and can also be determined by DLS [40,41]. The zeta potential value plays a pivotal role in predicting the stability of SLBNs during storage. Generally, a zeta potential value of approximately −30 mV is considered sufficient for stabilizing SLBNs [42,43].

**Figure 6:**
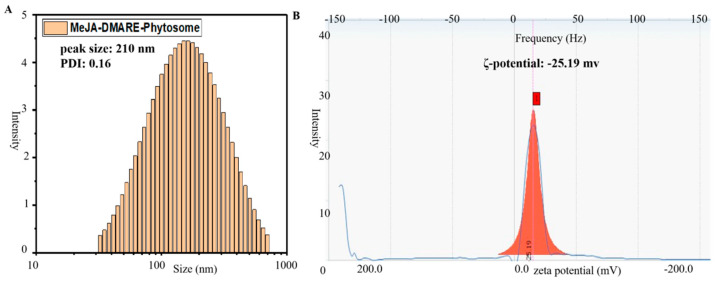
Images illustrating (**A**) the size distribution and (**B**) the zeta potential distribution of the phytosomes loaded with MeJA-DMARE.

In summary, the excellent stability of the prepared phytosomes is highlighted by their small particle size (210 nm), uniform size distribution, low PDI value (0.16), and superior zeta potential (−25.19 mV), as shown in Figure 6.

#### 3.2.4. Potential Interactions between MeJA-DMARE and Phospholipids

**Figure 7:**
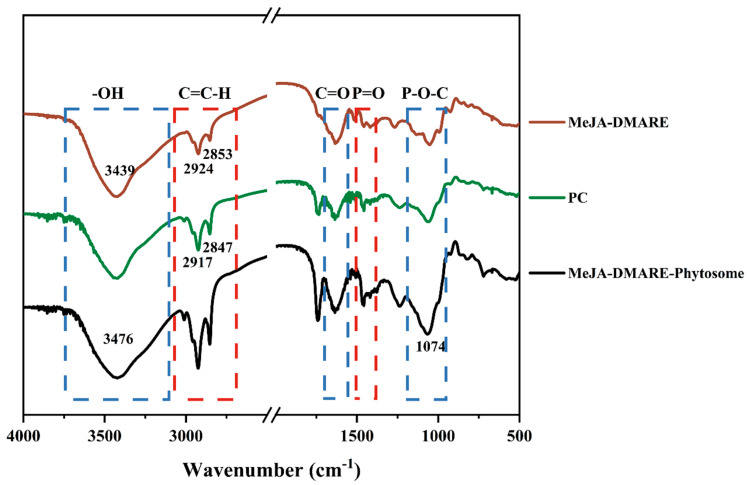
FTIR spectra of MeJA-DMARE phytosomes, PC, and MeJA-DMARE.

FTIR analysis was employed to investigate potential interactions between polyphenols of MeJA-DMARE and phospholipids. Infrared (IR) spectra were captured within the 4000 to 500 cm^−1^ range. These IR spectra were then analyzed to identify the functional groups associated with polyphenols, phosphatidylcholine, and phytosomes, with their corresponding wavenumbers (cm^−1^) outlined in Figure 7. The distinction in the fingerprint region (below 1500 cm^−1^) signifies the creation of a novel phytosome complex. The spectra displayed a peak at 3439 cm^−1^, indicating the presence of aliphatic alcoholic (−OH) groups typically found in polyphenolic compounds [31]. The spectra revealed the presence of long-chain fatty acid bands from the phospholipid molecule at 2932 and 2957 cm^−1^, further indicating the formation of phytosomes [44]. When comparing the spectra of PC with those of phytosomes, it was observed that in the phytosomal structure, the peaks corresponding to phospholipid bonds P=O (1463 cm^−1^) and P-O-C (1055 cm^−1^) in the choline residues had shifted to 1469 cm^−1^ and 1074 cm^−1^, respectively. Unlike the MeJA-DMARE, the phytosome spectrum displayed a synergistic effect on its constituents. For example, the characteristic signals associated with the O-H (3439 cm^−1^) in phenolics and C=O (1644 cm^−1^) in polar groups of phospholipids merged into new peaks at 3476 cm^−1^ and 1652 cm^−1^ in phytosomes, respectively. Therefore, it is reasonable to infer that hydrogen bonding has occurred between the O–H groups of MeJA-DMARE and the polar segments of phospholipids. As a result, these relatively modest intermolecular forces could be a significant contributor to the formation of the MeJA-DMARE phytosomes.

#### 3.2.5. In Vitro Stability

The stability of the fabricated phytosomes under in vitro conditions was investigated using a range of solutions including distilled water, 20 mM glycine–HCl buffer (pH 2.0), citrate–sodium citrate buffer (pH 5.0), sodium phosphate buffer (pH 7.0), Tris-HCl buffer (pH 8.0), 5% sodium chloride, 10% sodium chloride, and 5% bovine serum albumin. A total of 900 μL of the solvents was combined with 100 μL of the phytosome solution, and the mixture was then incubated at 37 °C. No significant wavelength shift was observed under all specified conditions, even after one month, with all wavelength changes falling within a 4 nm range. Our findings confirm that MeJA-DMARE phytosomes remain intact, indicating outstanding in vitro stability in biological solutions at physical pH levels.

### 3.3. Cytotoxicity

**Figure 8:**
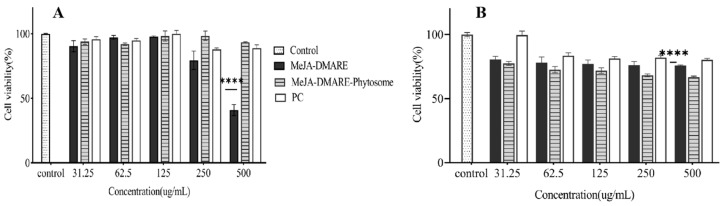
Cytotoxicity of MeJA-DMARE, MeJA-DMARE phytosomes, and PC. (**A**) Cytotoxicity in RAW 264.7 cells. (**B**) Cytotoxicity in A549 lung cancer cells. The figure displays the average ± standard deviation based on three separate experiments. Differences from the control are marked as highly significant with **** *p* < 0.0001.

Cytotoxicity has become the preferred screening test and a key parameter for assessing the toxicity of medical devices, mainly because of its ease of use, speed, high sensitivity, and ability to prevent harm to cells and animals from toxicological testing [45]. The MTT assay is a rapid and colorimetric technique used to swiftly assess cell growth and toxicity by quantifying cellular metabolism or functionality [46]. The MTT assay was utilized to gauge the toxicity of MeJA-DMARE and MeJA-DMARE phytosome against RAW 264.7 and A549 lung cancer cell lines. Based on our research (Figure 8), MeJA-DMARE phytosomes and PC exhibited lower cytotoxicity and high cell viability in RAW 264.7 cell lines up to 500 μg/mL compared to the MeJA-DMARE group. Moreover, MeJA-DMARE displayed increased cytotoxicity at concentrations over 250 μg/mL, resulting in a 35% reduction in cell proliferation. Consequently, we employed the concentrations of 125 μg/mL and 250 μg/mL for subsequent experiments. In contrast, MeJA-DMARE and PC demonstrated low cytotoxicity and high cell viability in A549 lung cancer cell lines until 500 μg/mL in contrast to the control. Conversely, MeJA-DMARE phytosomes exhibited cytotoxic effects at concentrations exceeding 250 μg/mL, leading to a notable 66–68% of cell proliferation. Given that 250 μg/mL is considered a safe concentration for cell growth in RAW cells, we selected it for A549 lung cell proliferation in our further research endeavors.

### 3.4. Enhanced Anti-Inflammatory Activities

#### 3.4.1. Inhibited NO and ROS Generation in RAW 264.7 Cells

NO’s physiological and pathophysiological functions within the respiratory system have been comprehensively reviewed [47]. NO plays a role in host defense within the bronchial epithelium and operates as an inflammatory mediator during pathological circumstances [48]. Excessive nitric oxide production occurs in abnormal conditions, including inflammatory bowel disease, arthritis, osteoporosis, and various inflammatory respiratory disorders. Consequently, restraining the overproduction of NO has emerged as a significant objective in treating inflammatory diseases [49].

**Figure 9:**
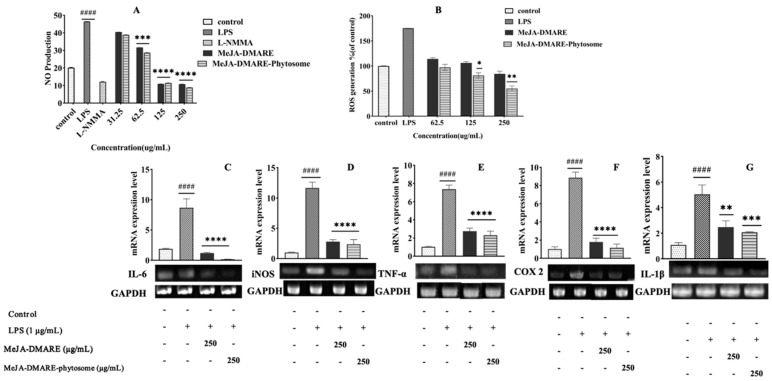
The anti-inflammatory effects on RAW 264.7 cells. (**A**) NO production; (**B**) ROS levels; (**C**–**G**) quantification of gene expression levels via qRT-PCR. Panels C through G correspond to the genes IL-6, iNOS, TNF-α, COX-2, and IL-1β, respectively. The graph displays the average ± standard deviation from three independent experiments. Significant differences between the LPS-treated group and the control group are denoted by #### *p*, and significant differences between the LPS-treated group and the experimental group are marked with * *p* < 0.05, ** *p* < 0.01, *** *p* < 0.001, and **** *p* < 0.0001. Original gel images can be found in Supplementary File S1.

We investigated the anti-inflammatory impact of MeJA-DMARE and MeJA-DMARE phytosome on RAW 264.7 cells. The cells were exposed to MeJA-DMARE and MeJA-DMARE phytosomes, followed by LPS (1 μg/mL) for 24 h. Our study employed L-NMMA, a well-known NO inhibitor, as a positive control. The NO production is markedly elevated in cells treated with LPS compared to the control group, as depicted in Figure 9A. However, in LPS-induced cells treated with MeJA-DMARE and MeJA-DMARE phytosomes, the NO production diminishes dose-dependently. Both MeJA-DMARE and MeJA-DMARE phytosomes showed marked and statistically significant reductions in NO synthesis at a concentration of 62.5 µg/mL, achieving decreases of 31.7% and 28.7%, respectively, versus cells treated with LPS alone. Within each treatment group, our synthesized phytosome displayed a superior ability to inhibit NO production compared to the plant extracts. This demonstrates the higher potential of our phytosome in terms of anti-inflammatory activities.

Macrophages assume vital functions in both initiating and perpetuating inflammation. In the context of endotoxemia and inflammation, macrophages become activated by LPS and cytokines. The activation of NADPH-oxidase in macrophages, induced by LPS, triggers the production of ROS. ROS is a mediator of cellular injury, playing a pivotal role in initiating cellular damage during endotoxemia. Additionally, there is a belief that ROS participates in controlling the expression of inflammatory genes by activating the NF-κB signaling pathway through redox-based mechanisms [50,51]. To investigate the inhibitory effects of MeJA-DMARE and MeJA-DMARE phytosomes on ROS generation triggered by LPS in RAW 264.7 cells, the cells were treated with varying concentrations of these compounds in the presence or absence of LPS for 24 h. As depicted in Figure 9B, a marked increase in ROS levels was observed in the LPS-exposed group. In contrast, the administration of MeJA-DMARE and MeJA-DMARE phytosomes to LPS-stimulated cells led to a progressive reduction in ROS production, following a dose–response pattern. Within each treatment group, our synthesized phytosome displayed a superior ability to inhibit ROS production in LPS-activated RAW 264.7 cells compared to the plant extracts. A notable reduction in ROS production was detected for MeJA-DMARE phytosomes and MeJA-DMARE at concentrations higher than 125 µg/mL. This demonstrates the higher potential of our phytosome in terms of anti-inflammatory activities.

As reported in our previous study, the MeJA-treated D. morbifera adventitious roots showed a heightened presence of a phenolic compound, 3,5-DCQA. This rise in concentration led to an amplified anti-inflammatory effect, as evidenced by the diminished output of NO and ROS in LPS-stimulated RAW cells. Nonetheless, compared to *D. morbifera* adventitious roots treated with MeJA, our synthesized phytosome demonstrated superior anti-inflammatory properties. Phytosomes represent modern formulations that enhance the bioavailability of hydrophilic phenolics. Phytomolecules have been noted for their improved absorption characteristics and enhanced lipid solubility, enabling them to traverse biological membranes effectively [52]. Previous reports have indicated that phytomolecules showed enhanced anti-inflammatory activities [53,54].

#### 3.4.2. Inhibition of Inflammation-Related Cytokines

Inflammation is a complex and evolutionarily conserved biological process triggered by the disruption of tissue homeostasis due to biological, physical, or chemical stimuli [55]. In response to these stressors, the innate and adaptive immune systems collaborate to initiate regulated inflammatory responses, aiming to manage tissue damage or combat pathogen invasions [56]. Nonetheless, while acute inflammation serves as a beneficial mechanism, especially when combating infectious agents, chronic inflammation is an undesirable occurrence that can eventually contribute to the development of inflammatory diseases such as arthritis, asthma, multiple sclerosis, inflammatory bowel disease, and atherosclerosis [57]. The expression of genes induced by LPS is controlled through a range of signaling pathways, including nuclear factor NF-κB, mitogen-activated protein kinases (MAPKs), and signal transducers and activators of transcription (STATs) [58]. NF-κB plays a significant role in the development of inflammatory diseases and in regulating the transcription of pro-inflammatory mediators such as iNOS, COX-2, TNF-α, and IL-1β [58]. Macrophages exhibit high inducible NOS (iNOS) expression, which is responsible for synthesizing NO [59]. Therefore, we investigated the impact of MeJA-DMARE phytosomes on the expression of these factors using RT-PCR and qRT-PCR. As shown in Figure 9C–G, in the group treated with LPS, there was a significant elevation in the mRNA expression of COX-2, TNF-α, iNOS, IL-6, and IL-1β (8.86-, 7.38-, 11.67-, 8.70- and 5.04-fold as compared to untreated cells). Nevertheless, the MeJA-DMARE phytosomes at 250 µg/mL reduced gene expression by 1.16-, 2.31-, 2.37-, 0.11-, and 2.08-fold compared to untreated cells. Within each treatment group, phytosomes showed better inhibitory effects of inflammation-related mRNA gene expression than MeJA-DMARE.

### 3.5. Enhanced Anti-Lung Cancer Activities

#### 3.5.1. Increased ROS Generation in A549 Cells

Cancer cells often exhibit an increase in the generation of ROS and a decrease in ROS scavenging [60]. Although excessive ROS levels can be toxic, sub-lethal ROS production plays a vital signaling role, especially in cancer, where ROS promotes activities like cell proliferation, migration, and invasion. Consequently, the induction of ROS accumulation in cancer cells is regarded as a novel therapeutic strategy aimed at selectively triggering apoptosis [61,62].

**Figure 10:**
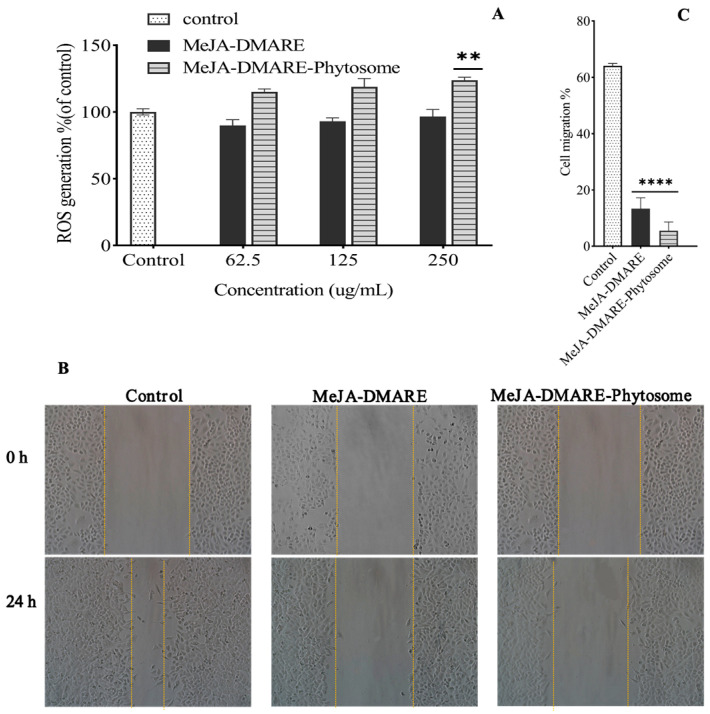
Anti-lung cancer effects. (**A**) MeJA-DMARE’s ability to enhance intracellular ROS generation. (**B**) Lung cancer cell migration levels. The blank space within the wounded area was measured using ImageJ 1.54j. (**C**) The blank space within the wounded area was measured using ImageJ. (**C**) The proportion of cell movement following a 24 h treatment versus the control. The graph shows the mean ± SD values in triplicates. Statistically significant changes from the control group are indicated by ** *p* < 0.01 and **** *p* < 0.0001.

According to our previous findings, MeJA-DMARE can promote ROS generation in A549 cells. Thus, we explored whether MeJA-DMARE phytosomes might induce ROS generation in the same cell lines. We used DCFH-DA to assess cellular and mitochondrial ROS levels. DCFH cannot easily cross cell membranes. When exposed to intracellular ROS, it can quickly transform into highly fluorescent 2,7-dichlorofluorescein [63]. The oxidative stress levels are indicated by fluorescence intensity. Treatment with MeJA-DMARE phytosomes at 250 and 500 µg/mL resulted in a dose-dependent increase in intracellular ROS generation in A549 cells. Within each treatment group, A549 cells treated with MeJA-DMARE phytosomes promoted more ROS generation than MeJA-DMARE (Figure 10A). Bioactive substances can induce apoptosis in cancer cells by increasing the buildup of ROS [64]. Our findings revealed that MeJA-DMARE phytosomes can promote ROS generation. Previous research has documented enhanced anticancer effectiveness of phytosomes compared to free polyphenols in lung cancer cell lines [65]. Therefore, our synthesized phytosomes, which induce elevated ROS production in cancer cells, have the potential to trigger multiple cell death pathways, ultimately restraining cancer progression. 

#### 3.5.2. Increased Inhibition of Lung Cancer Cell Migration

The concerted migration of cells, characterized by their collective movement in sheets, strands, clusters, or ducts rather than individually, is a distinctive aspect of tissue remodeling processes observed in embryonic development, wound healing, and cancer progression. This type of migration involves the use of actin- and myosin-dependent protrusions and is directed by external chemical and mechanical cues, which is analogous to the guidance mechanisms employed by single migratory cells [66]. It remains a formidable challenge to hinder cancer cells from migrating and invading various organs in cancer therapy. A wound closure test was conducted to check the migration (%) levels of A549 lung cancer cells. The migration levels were evaluated before and after treatment with samples at 250 µg/mL. Our investigation revealed a reduction in migratory cells after treatment with MeJA-DMARE and MeJA-DMARE phytosomes, indicating that both inhibit the lateral movement of A549 cells. Notably, MeJA-DMARE phytosomes demonstrated stronger inhibition compared to MeJA-DMARE. Thus, our study suggests that MeJA-DMARE phytosomes effectively impede the proliferation and metastasis of A549 cells in vitro (Figure 10B,C). The ability of MeJA-DMARE phytosomes to inhibit tumor growth could signal their potential in lung cancer therapy.

#### 3.5.3. Apoptotic Gene Expression in Lung Cancer Cells

**Figure 11:**
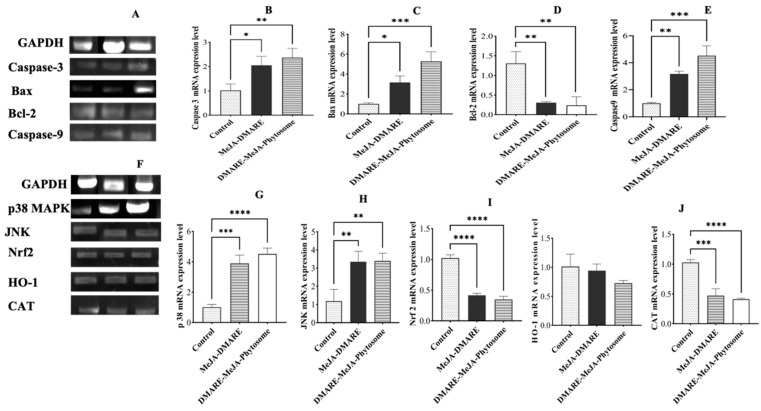
Apoptosis-related gene mRNA expression levels in A549 cells via the Bcl-2 and MAPK pathways. (**A**,**F**) The mRNA expression bands via from agarose gel electrophoresis. (**B**–**E**,**G**–**J**) Quantification of gene expression levels via qRT-PCR. Genes from (**B**–**E**,**G**–**J**) include Caspase 3, Bax, Bcl-2, Caspase 9, p38 MAPK, JNK, Nrf-2, HO-1, and CAT. Statistically significant differences in triplicate vs. the control group are denoted by * *p* < 0.05, ** *p* < 0.01, *** *p* < 0.001, **** *p* < 0.0001, while ns denotes no significant difference. Original gel images can be found in Supplementary File S1.

Bcl-2 is an anti-apoptotic protein produced in response to DNA damage. It is a critical player in controlling cell death and acting as an indicator of genotoxic stress within the cell [67,68]. As depicted in Figure 11A–E, MeJA-DMARE phytosomes significantly reduced Bcl-2 expression by 0.24-fold. In contrast, they significantly increased the mRNA expression of Caspase 3, Caspase 9, and Bax by 2.37, 4.53, and 6.26 times, respectively.

On the other hand, heme oxygenase-1 (HO-1) is well known for its strong induction in response to various stressors and many cancer chemo-preventive agents [69]. However, in human cancers, the overexpression of HO-1 provides cancer cells with a growth advantage and makes them more resistant to chemotherapy and photodynamic therapy [70]. Nrf2 is a pivotal transcription factor in defending against oxidative stress and activating Nrf2 is a potent strategy for preventing cancer induced by exposure to environmental carcinogens [71]. This activation is believed to be controlled by several upstream signaling kinases, including mitogen-activated protein kinases (MAPKs such as p38 and JNK), which regulate Nrf2/ARE activity [72]. As illustrated in Figure 11F–K, the mRNA expression levels of p38 MAPK and JNK were at their lowest in the control group. However, MeJA-DMARE phytosomes significantly up-regulated these expressions in A549 lung cancer cells (p38 MAPK and JNK increased by 4.52 and 3.40 times, respectively). Furthermore, MeJA-DMARE phytosomes were able to significantly down-regulate these expressions in A549 lung cancer cells (Nrf2, HO-1, and CAT decreased by 0.37, 0.79, and 0.44 times, respectively). Within each treatment group, phytosomes showed better stimulatory effects of apoptosis-related mRNA gene expression than MeJA-DMARE. Nevertheless, the relevant protein levels are best quantified by some technology for further confirmation. 

Why do phytosomes exhibit better biological properties and hold improved delivery efficiency compared to phytochemicals alone? Phytosomes’ phospholipid bilayer facilitates medication administration by forming a lipid–lipid interface between the cell membrane and the carrier, allowing phytochemicals to diffuse into the cell. Additionally, specific ligands can be used to modify the surface of these carriers, making them active targets. Phytosomes can improve the effectiveness of lipophilic medication diffusion through the small intestine brush barrier. Drugs tend to agglomerate and hydrolytic digestion can impede their regulated and sustained release into the bloodstream. Phytosomes include phosphatidylcholine, which forms a monolayer in the gastrointestinal tract to avoid drug aggregation [73]. However, the exact molecular mechanisms of phytosomes are not yet fully understood. It deserves to be made clear how phytosomes deliver drugs into target tissues effectively.

## 4. Conclusions

MeJA-DMARE phytosomes have demonstrated remarkable efficacy in enhancing the bioactivity and pharmacological attributes of MeJA-DMARE, including its potential as an anti-inflammatory and anti-lung cancer agent, all while exhibiting low toxicity. Furthermore, MeJA-DMARE phytosomes can enhance the solubility and permeability of active compounds across biological membranes, thereby maximizing bioavailability. These findings suggest that MeJA-DMARE phytosomes hold promise as a dependable option for optimizing drug dosages in lung cancer therapy.

## Figures and Tables

**Table 1:** Entrapment efficiency and loading content of MeJA-DMARE phytosomes.

Method	Formulation	Entrapment Efficiency (%)	Loading Capacity (%)
Co-solvency	A1 (1:1)	56.89 ± 3.09	51.71 ± 3.29
	A2 (1:2)	71.19 ± 4.88	62.91 ± 3.11
	A3 (1:3)	65.57 ± 3.49	61.85 ± 0.21
Thin-layer film	B1 (1:1)	43.14 ± 2.86	36.50 ± 3.64
	B2 (1:2)	79.98 ± 1.45	69.17 ± 0.14
	B3 (1:3)	57.90 ± 1.87	54.98 ± 4.90

## Data Availability

The data are contained within the article.

## Supplementary Materials

The following supporting information can be downloaded at: https://www.mdpi.com/article/10.3390/biom14101273/s1, File S1: Original gel images.

## Abbreviations

DMARE = *Dendropax morbifera* adventitious root extract; MeJA = methyl jasmonate; 3,5-DCQA = 3,5-Dicaffeoylquinic Acid; MTT = 3-(4,5-dimethylthiazol-2-yl)-2,5-diphenyltetrazolium bromide; LPS = lipopolysaccharide; L-NMMA = NG-Monomethyl-L-arginine, monoacetate salt; NO = nitric oxide; ROS = reactive oxygen species; FE-SEM = field emission scanning electron microscopy; FE-TEM = field emission transmission electron microscopy; FTIR = Fourier transform infrared spectroscopy; DLS = dynamic light scattering; PDI = diameter and polydispersity index; PC = soy phosphatidylcholine.
