# Supplementary material for: Preparing a Phytosome for Promoting Delivery Efficiency and Biological Activities of Methyl Jasmonate-Treated Dendropanax morbifera Adventitious Root Extract (DMARE)
Source: Biomolecules. 2024 Oct 10;14(10):1273. doi: 10.3390/biom14101273 (PMC11505992; doi:10.3390/biom14101273)
Supplement: Supplementary file 1 [file biomolecules-14-01273-s001.zip › biomolecules-3175317-supplementary.pdf]

Supplementary materials-biomolecules-3175317

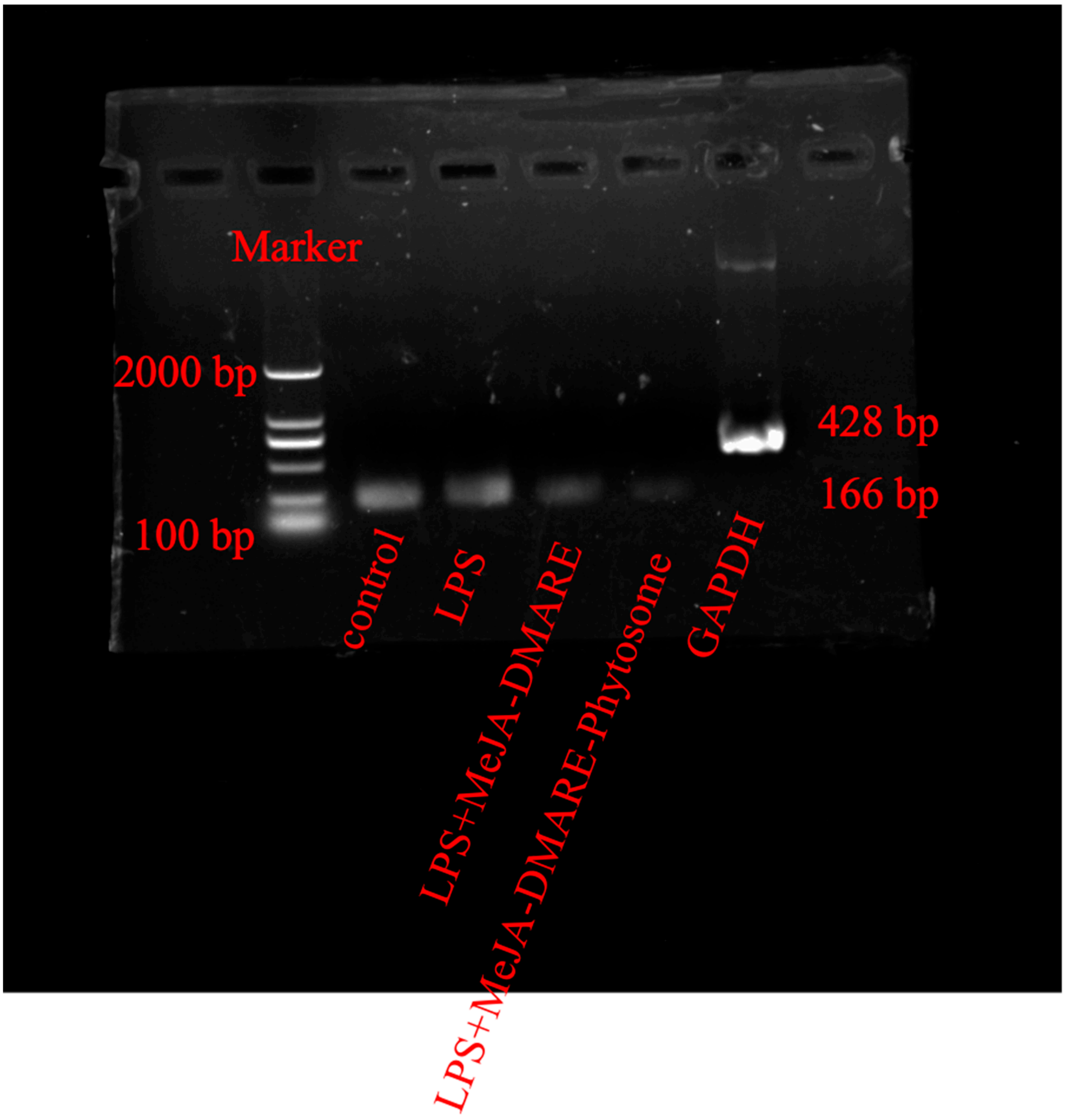

mRNA expression of *IL-6* gene in agarose gel electrophoresis bands.

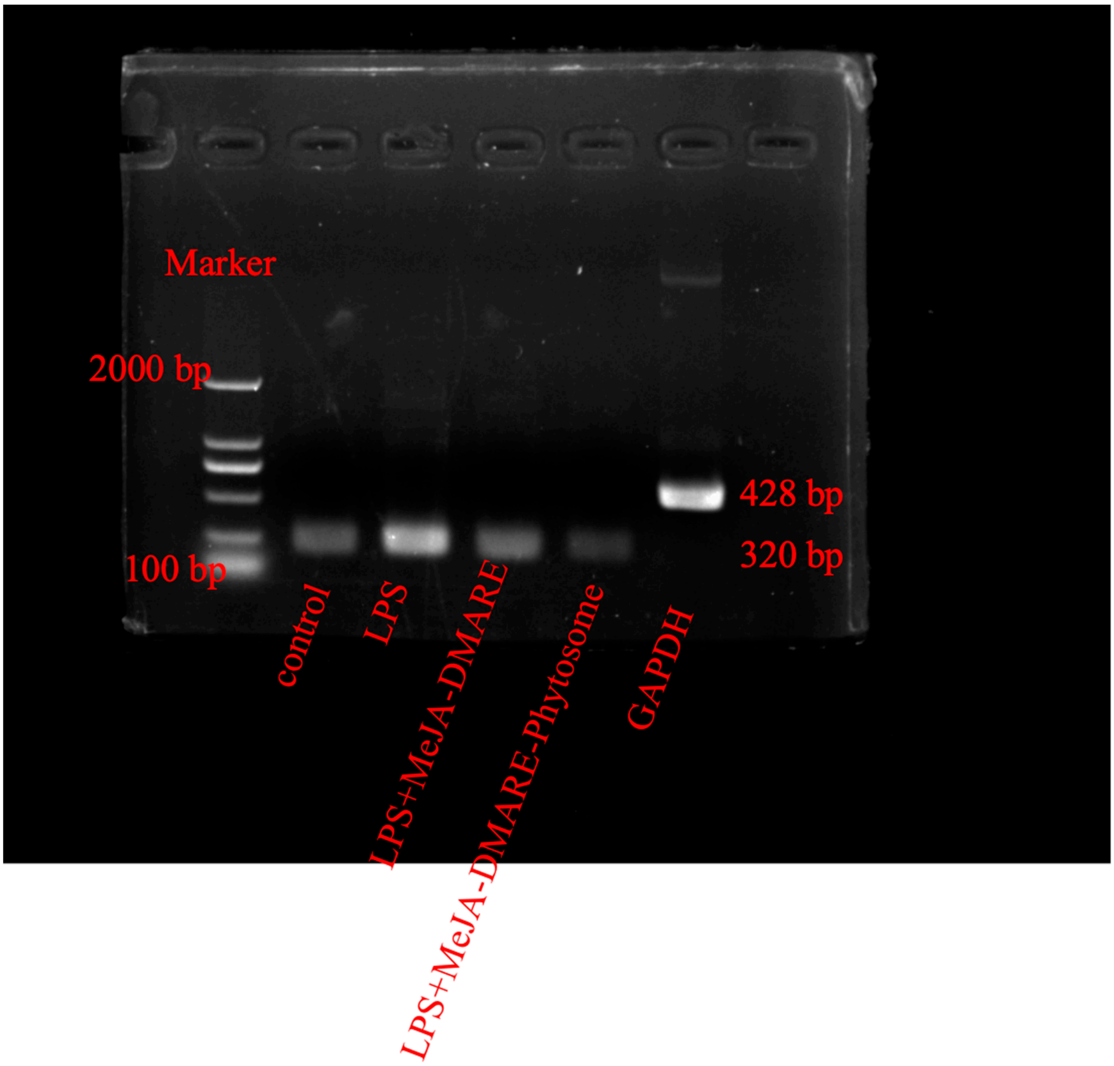

mRNA expression of *iNOS* gene in agarose gel electrophoresis bands.

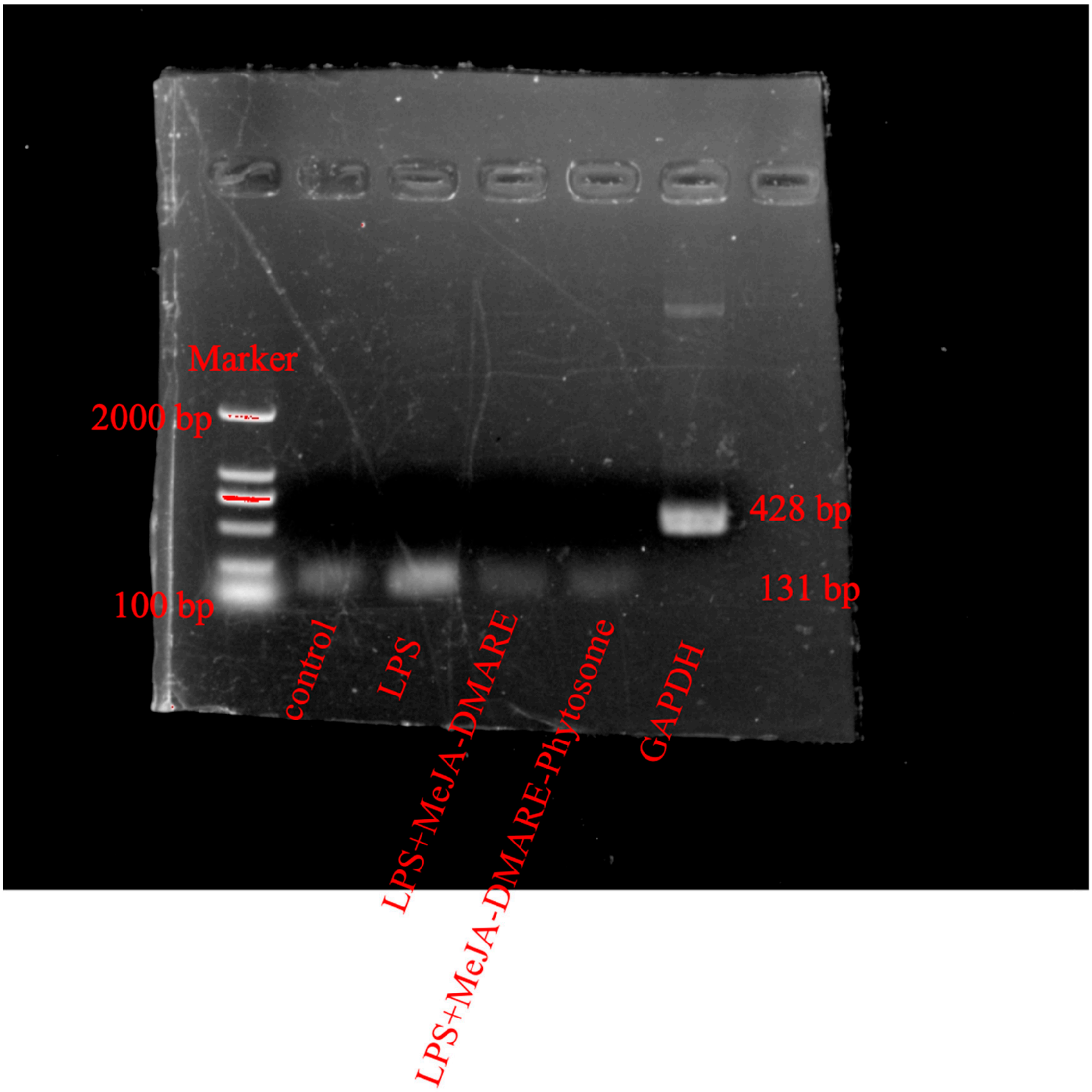

mRNA expression of *TNF-α* gene in agarose gel electrophoresis bands.

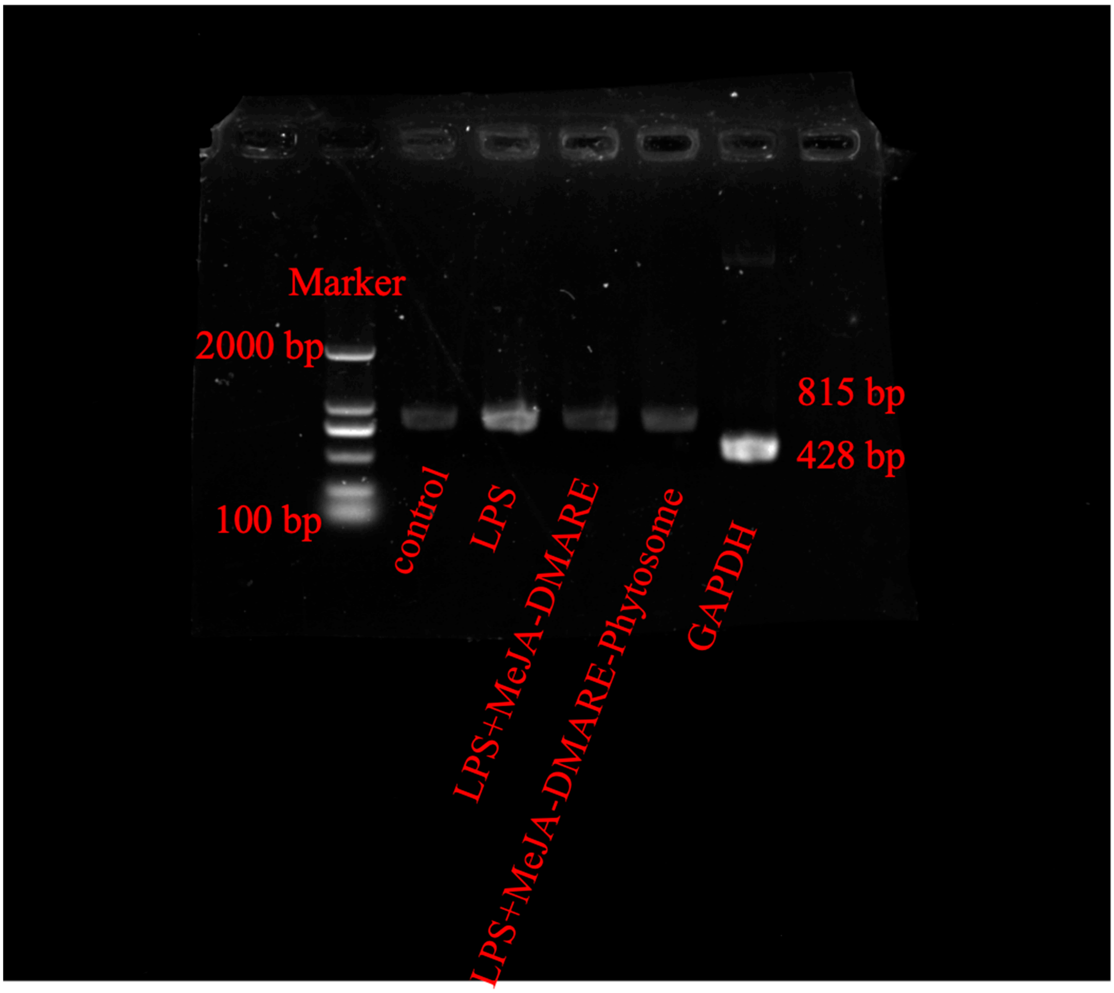

mRNA expression of *COX-2* gene in agarose gel electrophoresis bands.

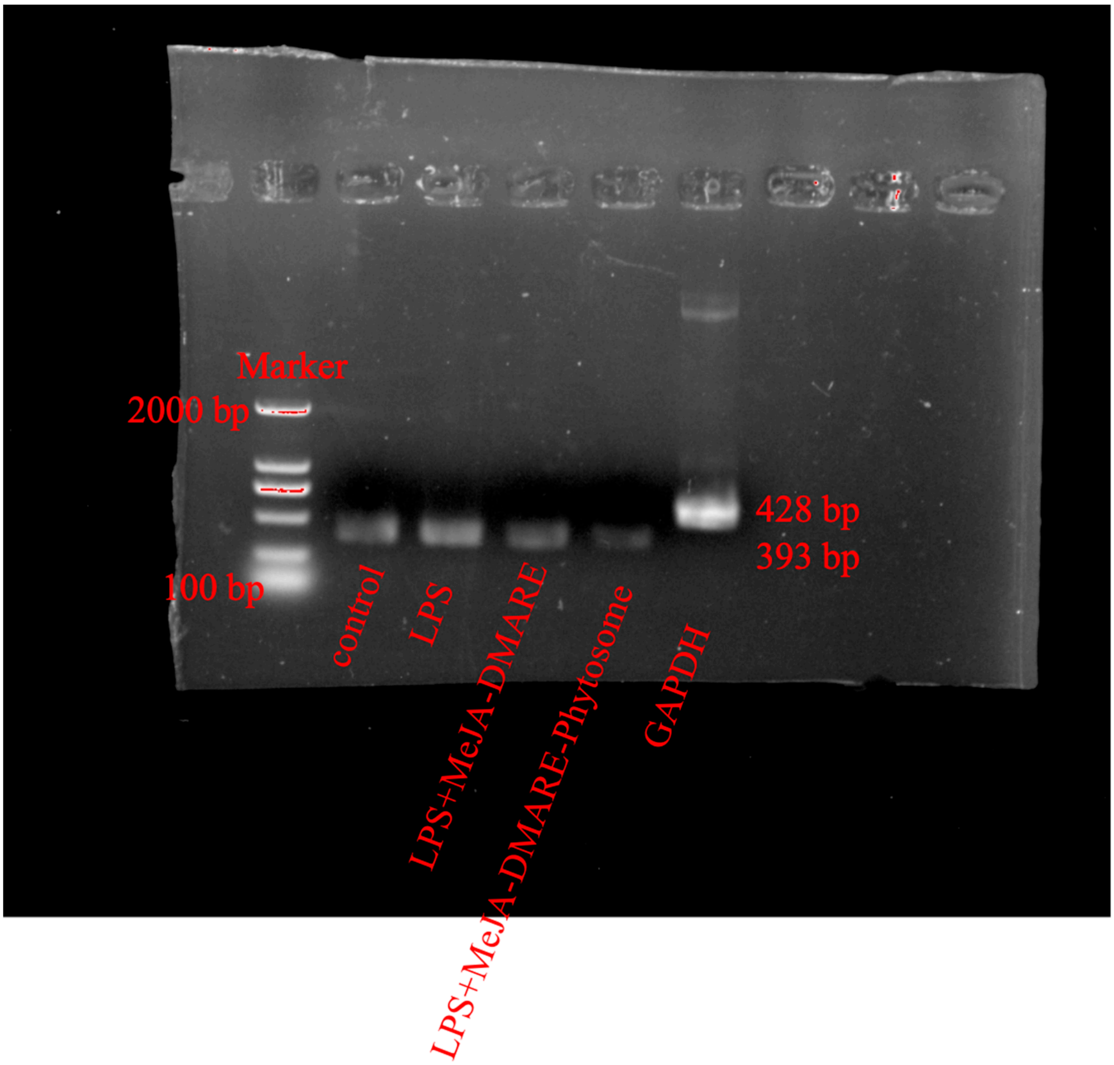

mRNA expression of *IL-1β* gene in agarose gel electrophoresis bands.

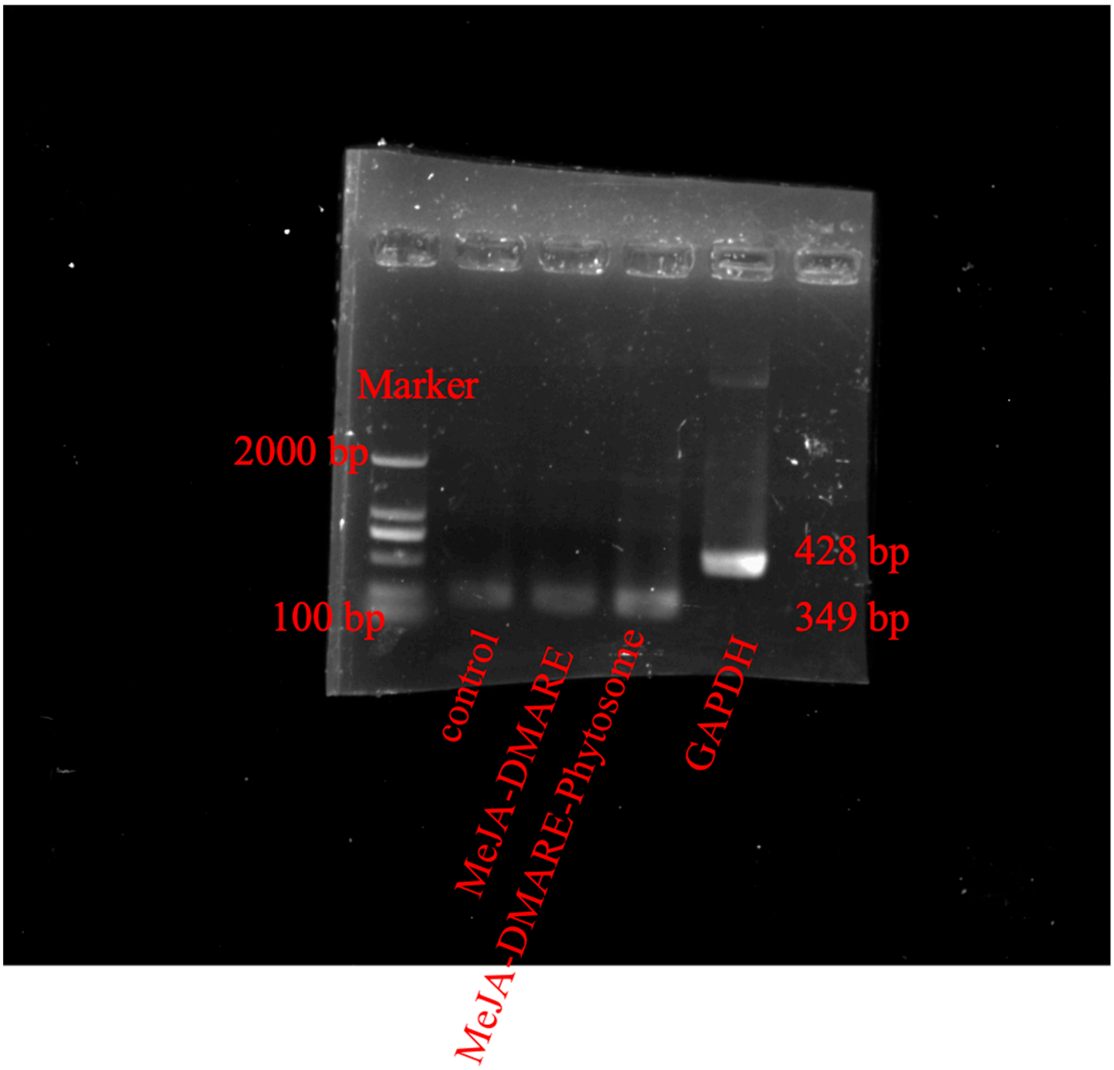

mRNA expression of *Caspase 3* gene in agarose gel electrophoresis bands.

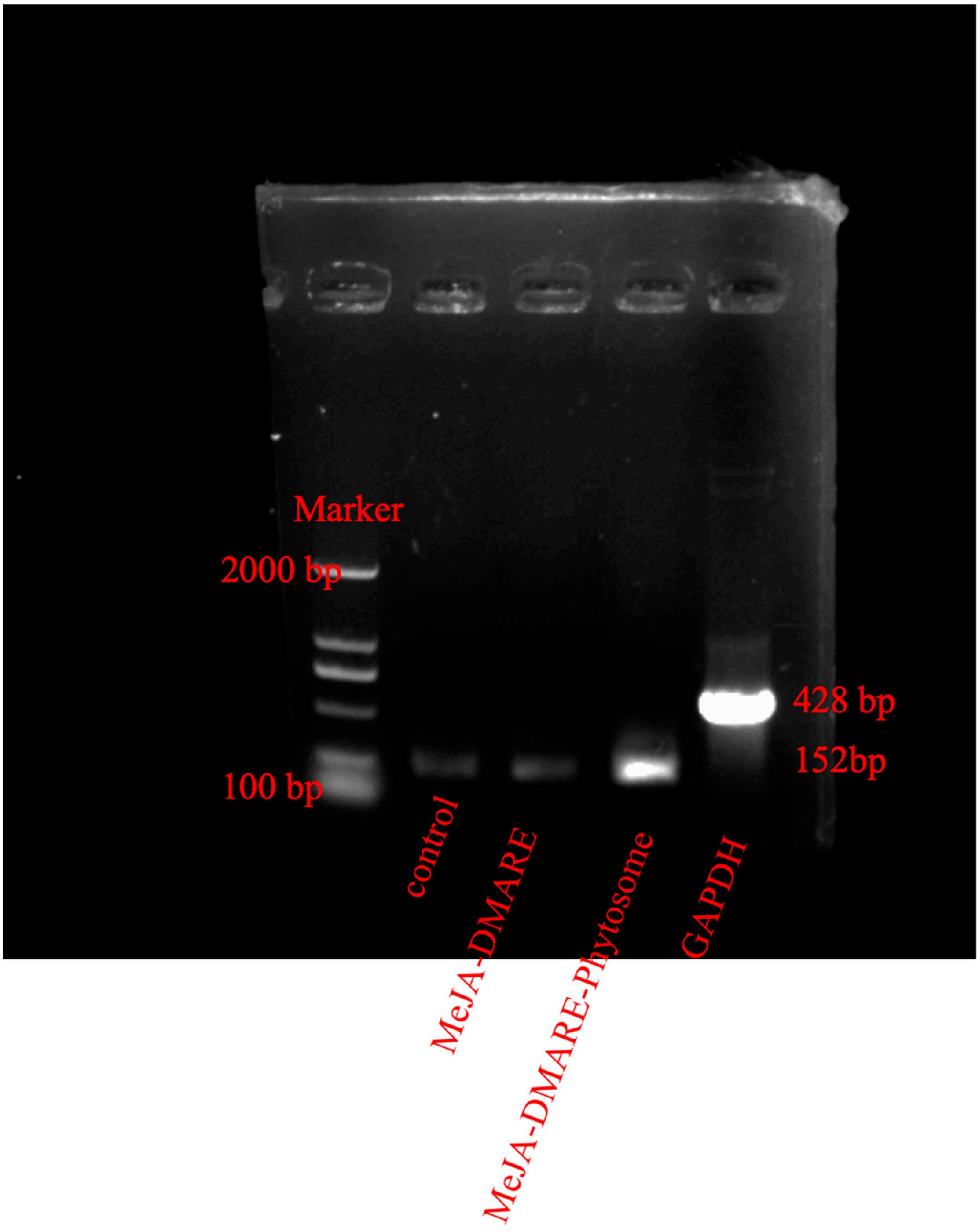

mRNA expression of *Bax* gene in agarose gel electrophoresis bands.

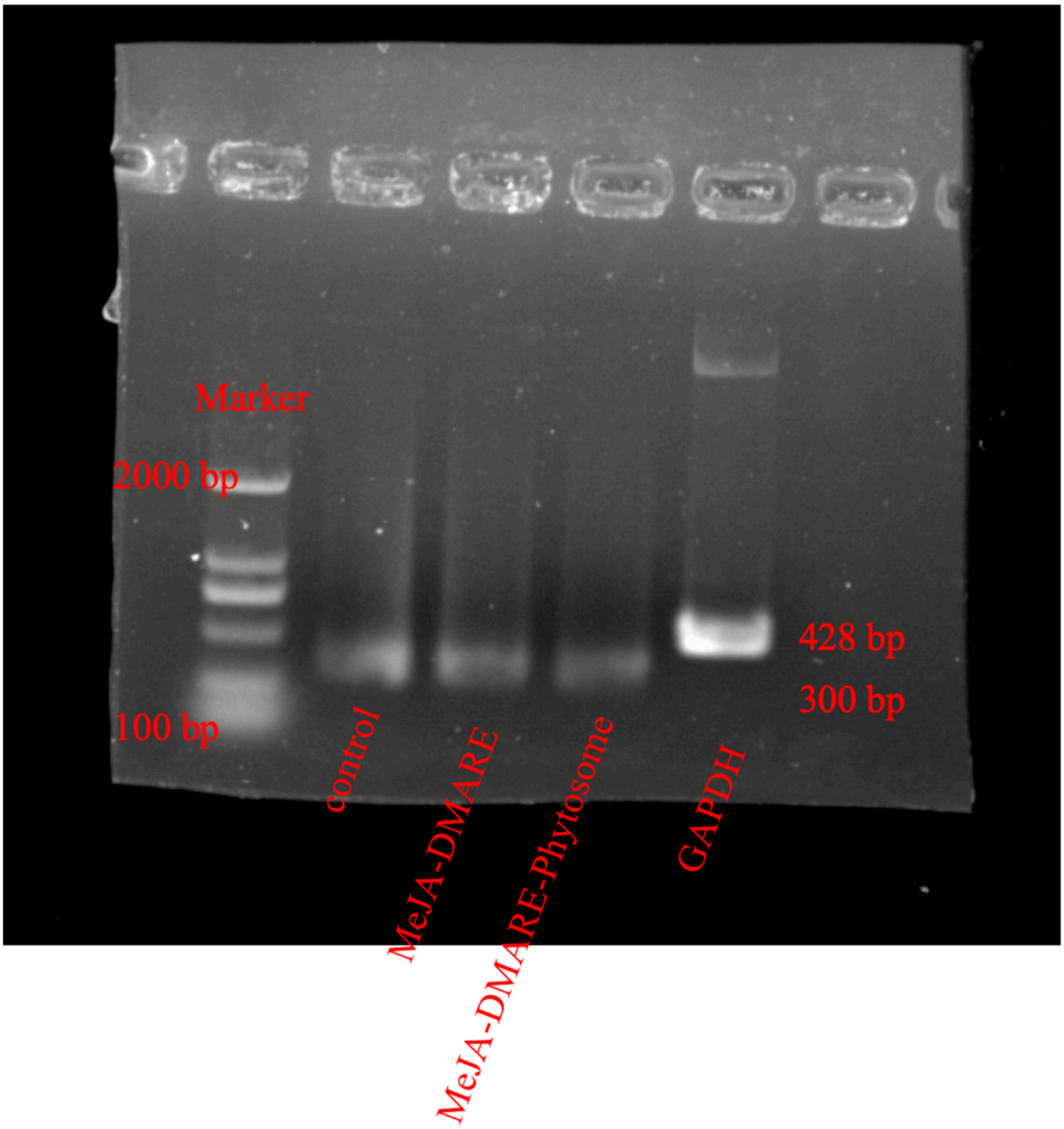

mRNA expression of *Bcl-2* gene in agarose gel electrophoresis bands.

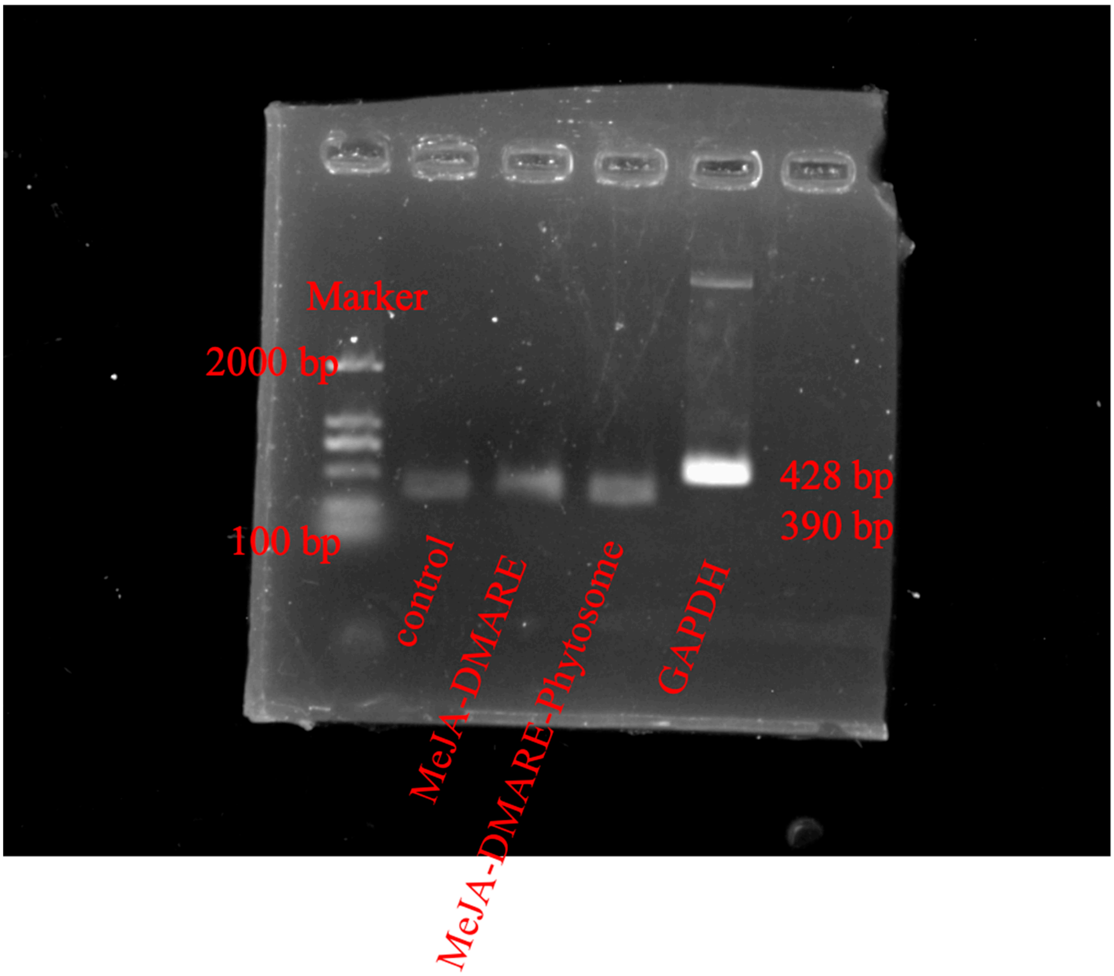

mRNA expression of *caspase 9* gene in agarose gel electrophoresis bands.

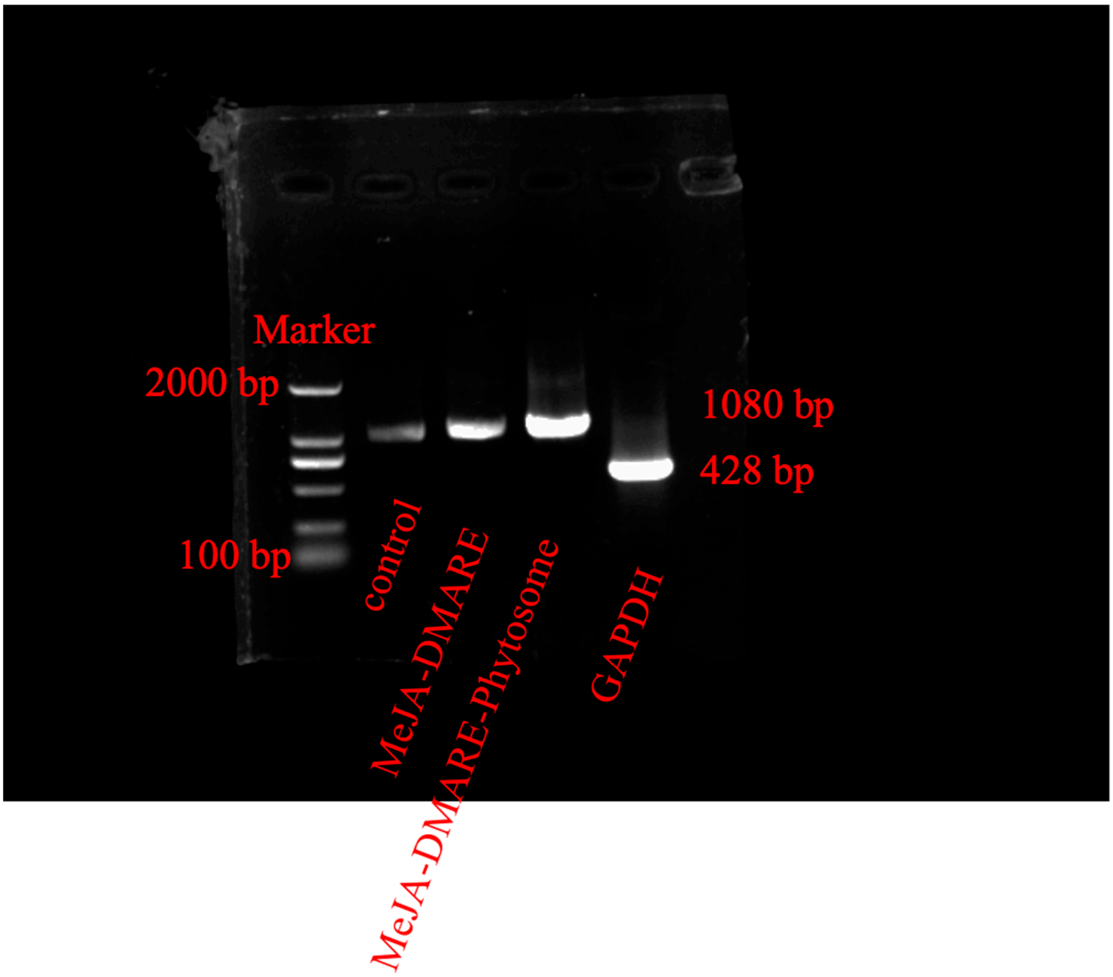

mRNA expression of *p 38 MAPK* gene in agarose gel electrophoresis bands.

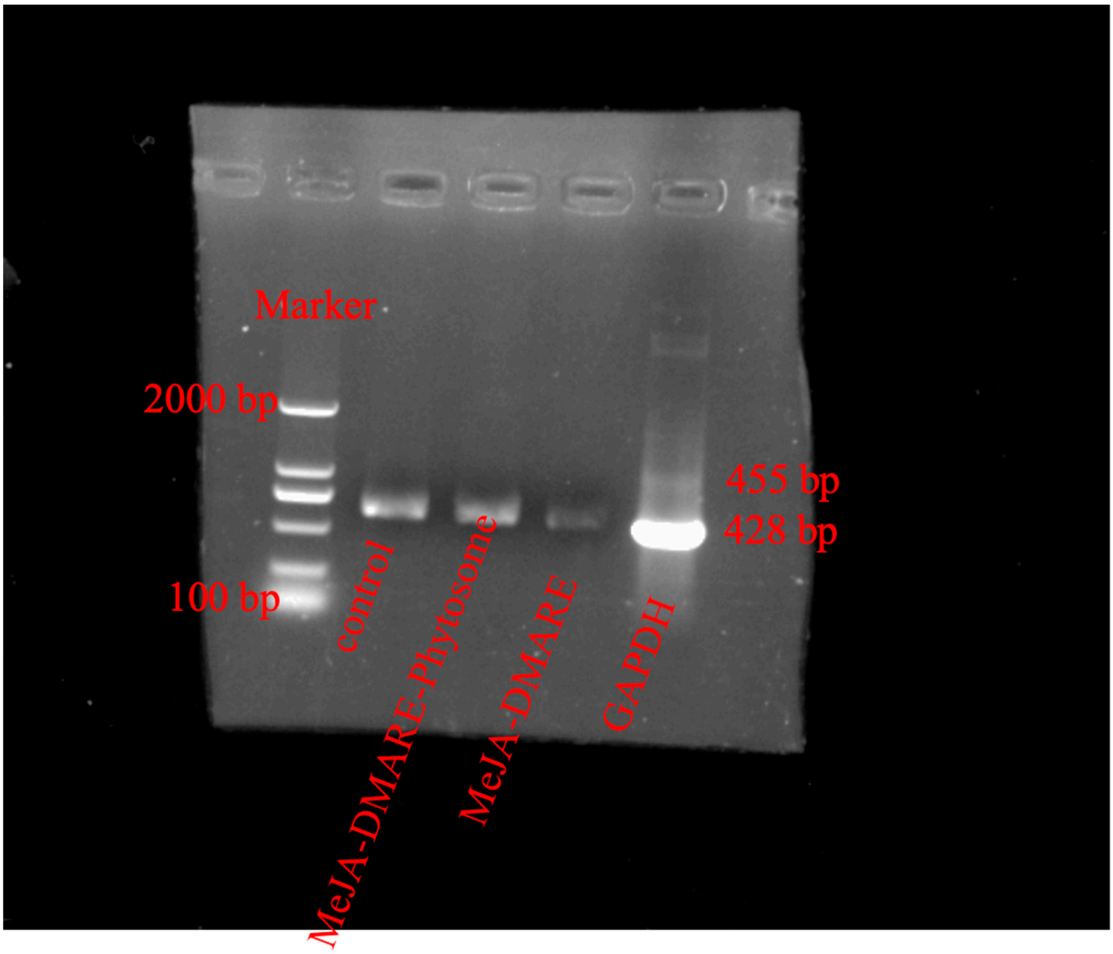

mRNA expression of *JNK* gene in agarose gel electrophoresis bands.

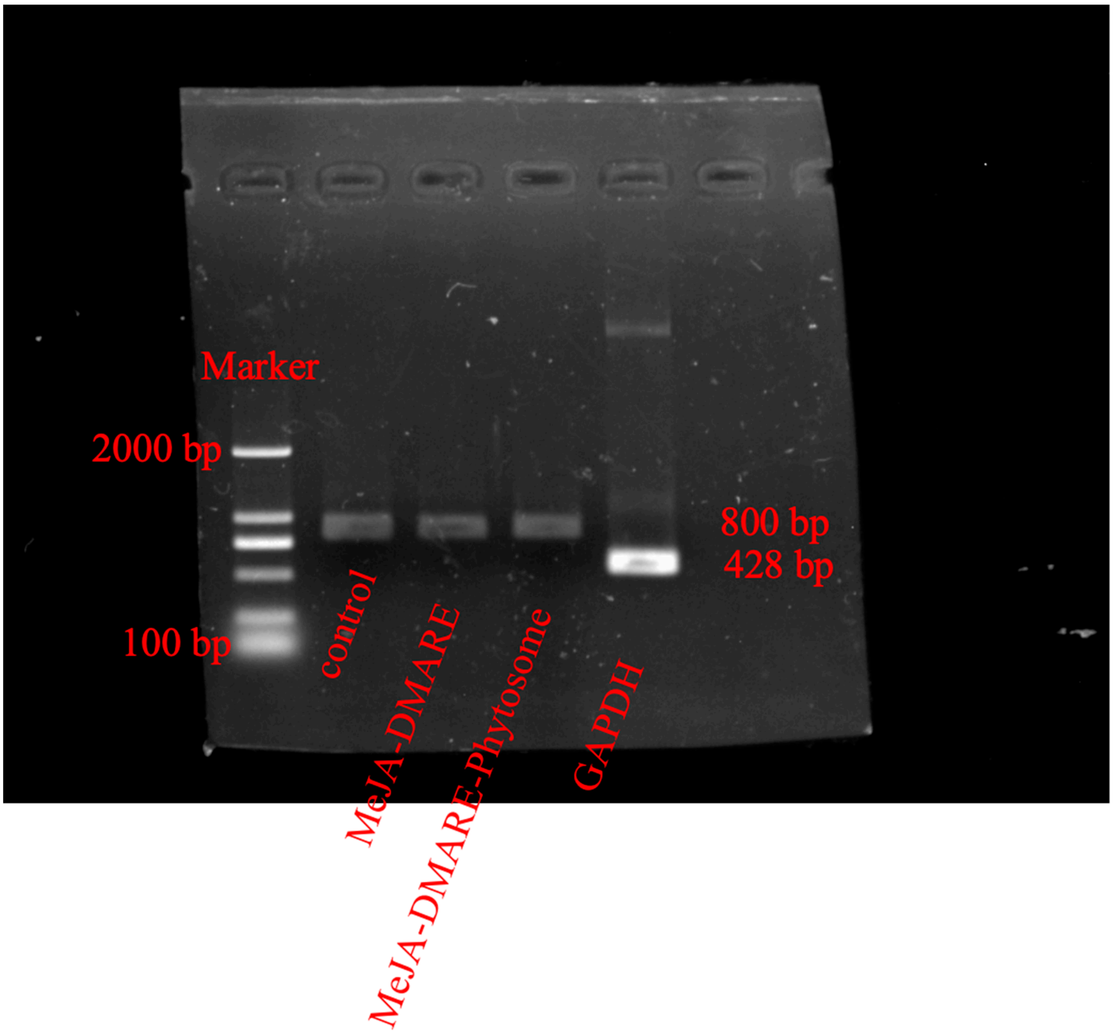

mRNA expression of *Nrf 2* gene in agarose gel electrophoresis bands.

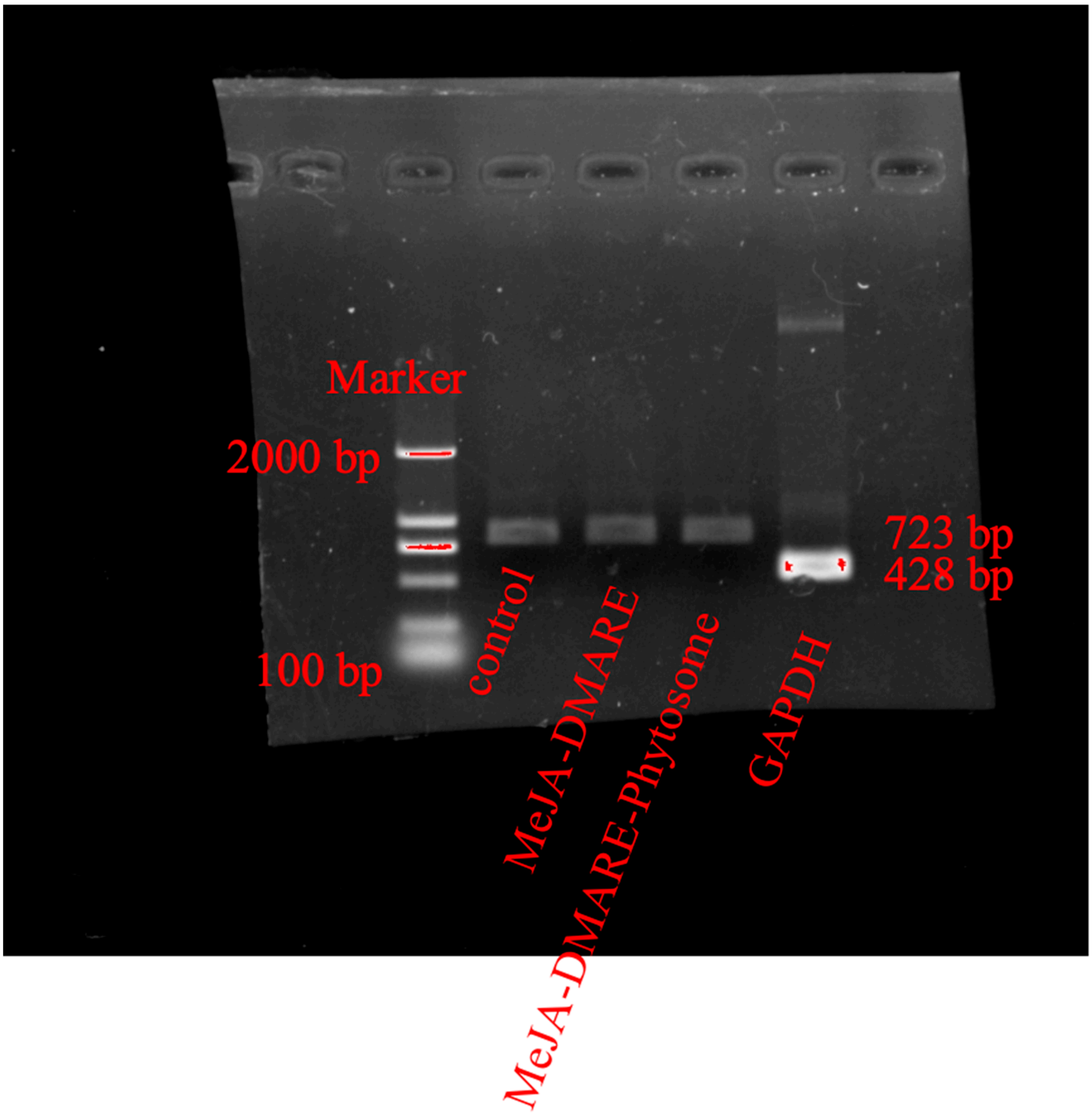

mRNA expression of *HO-1* gene in agarose gel electrophoresis bands.

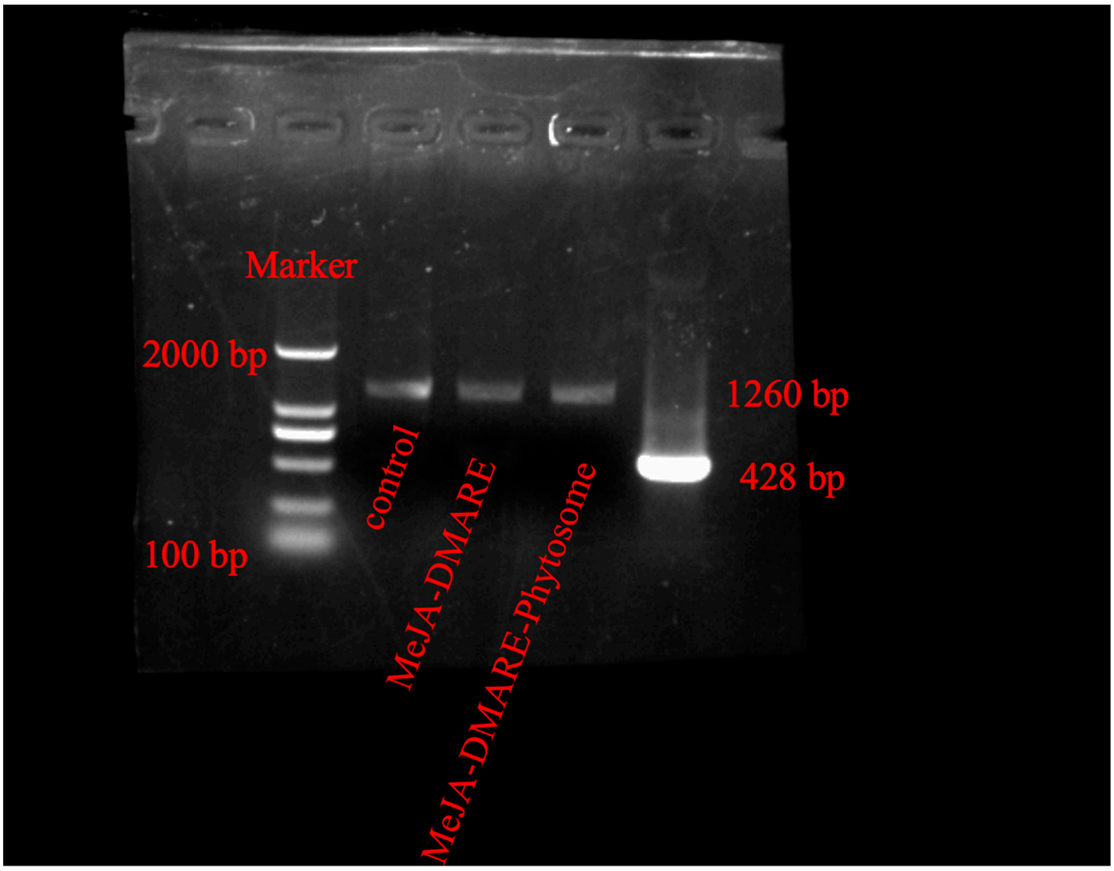

mRNA expression of *CAT* gene in agarose gel electrophoresis bands.
